# Mannose Targeting and Hydrophobic Tuning of Polycationic Vectors for Efficient Immunostimulatory CpG Delivery

**DOI:** 10.1021/acsanm.5c03883

**Published:** 2025-11-17

**Authors:** Federica Bellato, Greta Bellio, Daniele Asnicar, Rosa Catania, Linda Pecchielan, Lara Marcenta, Marco Zanon, Anna Cielo, Marica Zainotto, Marco Pirazzini, Alberta Ferrarini, Giuseppe Mantovani, Francesca Mastrotto

**Affiliations:** † Department of Pharmaceutical and Pharmacological Sciences, 9308 University of Padova, Via F. Marzolo 5, 35131 Padova, Italy; ‡ Department of Chemical Sciences, University of Padova, Via F. Marzolo 1, 35131 Padova, Italy; § School of Pharmacy, 6123 University of Nottingham, Nottingham NG7 2RD, U.K.; ∥ School of Chemistry, 4468 University of Leeds, Leeds LS2 9JT, U.K.; ⊥ Department of Biomedical Sciences, University of Padova, Via Ugo Bassi 58/B, 35131 Padova, Italy

**Keywords:** cationic polymers, glycoplex, mannose receptor, CpG, cancer vaccination

## Abstract

The efficacy of nucleic acid-based therapeutics is often hindered by nuclease degradation and poor cellular uptake. To address these challenges, the complexation with cationic polymers to form polyplexes has been increasingly investigated. In our previous work, we developed a platform technology composed of a mannosylated block for targeting dendritic cells (DCs) via endocytic mannose receptor (CD206), an agmatinyl block for nucleic acid condensation in diblock copolymers (M_15_-*b*-A_12_, M_29_-*b*-A_25_, and M_58_-*b*-A_45_), elongated with a poly­(butyl acrylate) block to promote endosomal escape in triblock copolymers (M_29_-*b*-A_29_-*b*-B_9_ and M_58_-*b*-A_52_-*b*-B_32_). We exploited these copolymers to efficiently target DCs for cancer vaccination by delivering plasmid DNA encoding tumor-associated antigens (TAAs), using ovalbumin (pOVA) as a model antigen. However, successful T-cell activation requires an antigen presentation on DCs as major histocompatibility complex (MHC)-antigen complexes, along with immune stimulation, making vaccine adjuvants essential. In this study, we utilized mannosylated cationic copolymers to deliver cytosine-phosphate-guanosine oligodeoxynucleotides (CpG ODN) as a vaccine adjuvant and tested their effect in conjunction with pOVA to further enhance immune activation. Cationic glycopolymers efficiently condensed single-stranded DNA (ssDNA), forming stable, predominantly spherical glycoplexes with sizes ranging from 20 to 40 nm, as assessed by transmission electron microscopy (TEM) analysis. These mannosylated complexes showed high internalization by CD206-expressing cells. Confocal laser microscopy studies revealed rapid nuclear localization mediated by M_58_-*b*-A_52_-*b*-B_32_ triblock copolymer and slower endosomal escape for M_58_-*b*-A_45_ diblock copolymer-based glycoplexes. Furthermore, for M_58_-*b*-A_45_ diblock copolymer-based complexes, codelivering CpG and pOVA in the same particles induced stronger DC activation compared to coadministration of glycoplexes containing CpG and glycoplexes containing pOVA. These provide a structure–activity relationship for this class of mannosylated cationic glycopolymers for nucleic acid delivery to DCs and underscore the synergistic benefits of codelivering CpG and nucleic acid encoding TAAs for DC activation.

## Graphical abstract

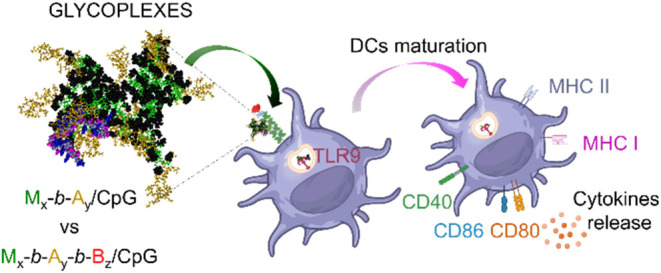

## 1 Introduction

Therapeutic nucleic acids (TNA) are widely recognized as one of the most rapidly growing classes of therapeutics, with many already approved and marketed.^1^ However, efficient delivery to the intended biological target in vivo still remains a major challenge for their clinical application^2^ due to their rapid degradation and/or limited ability to cross biological barriers, including cell membranes.^3^ To overcome these challenges, therapeutic nucleic acids are often formulated as complexes with oppositely charged (macro)­molecules, including cationic lipids and polymers. Among these, cationic polymers have emerged as suitable noncovalent complexing agents for a variety of TNA payloads. These polymers can be tailored in terms of composition, architecture (e.g., branched, linear, or dendritic^4^), size, and charge to electrostatically bind the negatively charged phosphate groups of TNAs. This interaction drives the formation of polyplexes, which protect the cargo from degradation, enhance cell association, and potentially facilitate endosomal escape.^5^

Over the years, an increasing understanding of the key features required for efficient transfection and specific cell recognition,^6^ combined with effort to minimize cytotoxicity, has led to the development of multifunctional polymers, for example, combining the ability to complex macromolecular drugs and to act as targeting agents.^7^ Moreover, their potentially lower production cost compared to viral vectors^8^ or lipid nanoparticles, along with their generally lower immunogenicity compared to viral vectors, and easy modification,^9,10^ have propelled the field of multifunctional polymers forward.

Recently, biocompatible polymers have been engineered to deliver nucleic acids to immune cells, potentially opening new avenues for immunotherapy, particularly in cancer treatment. Bellato et al.^11^ demonstrated the efficient delivery of plasmid DNA encoding tumor-associated antigens (TAAs) to dendritic cells (DCs) via mannosylated polycations, leading to specific CD8^+^ T-cell activation against tumors in vivo. Similarly, Zhang et al.^12^ used cationic poly­(β-amino ester) (PbAE) conjugated to dimannose moieties through a polyglutamic acid spacer to deliver specific mRNA and trigger an antitumor response by reprogramming macrophages into a cytotoxic and pro-inflammatory phenotype. Additionally, Kabanov and co-workers^13^ utilized two cationic block copolymers (PEG-*b*-PLL and PEG-*b*-pAsp) that were further functionalized with a single mannose residue as a targeting agent, for pDNA complexation. The targeted polymer showed enhanced transfection efficiency in macrophages compared to its nontargeted counterpart.

Thus, by delivering a range of nucleic acids—e.g., pDNA, PNA, mRNA, siRNA, miRNA, and CpG—polyplexes formed from functional polymers have the potential to restore immune cell recognition and activation against cancer cells that are suppressed within the tumor microenvironment.^14^ One emerging strategy for achieving this is to deliver nucleic acids encoding TAAs to antigen-presenting cells (APCs), which can present TAAs via major histocompatibility complex (MHC) class I and II molecules, stimulating CD8^+^ and CD4^+^ T cell responses against cancer cells. This approach triggers both an immediate immune response and long-term immune memory.^15^

Among nucleic acid-based therapeutics, synthetic single-stranded oligodeoxynucleotides (ODNs) containing unmethylated cytosine-phosphate-guanosine (CpG) motifs are of particular interest. CpG ODNs trigger an innate immune response via Toll-like receptor 9 (TLR9), since they share sequence analogous to bacterial DNA, thus activating the same immunostimulatory pathway.^16^ Hence, CpG molecules stimulate B cells and plasmacytoid dendritic cells (pDCs) and activate both innate and adaptive immune responses, making them promising for the treatment of infectious diseases, cancers, and allergies.^17^ However, like other nucleic acids, CpG ODNs are rapidly degraded by DNases. Efforts to stabilize CpG ODNs via chemical modifications have raised concerns about potential toxic side effects of the resulting modified oligodeoxynucleotides,^18^ prompting the development of nanocarriers to improve delivery, protect against degradation, and enhance cellular uptake.

To achieve robust cellular activation, efficient delivery of CpG ODNs to target antigen-presenting cells is paramount. Leveraging the high expression of the mannose receptor (MR, CD206)^19^ on these cells, mannosylated polyplexes have been successfully employed for targeting dendritic cells (DCs) and macrophages, paving the way for the exploitation of the CD206-mannose axis as part of vaccination strategies.^20^

In this work, we utilized a library of diblock cationic copolymers displaying a mannosylated block (M_*x*_) for targeting CD206 expressed on antigen-presenting cells, and a cationic agmatinyl block (A_*y*_)—M_15_-*b*-A_12_, M_29_-*b*-A_25_, and M_58_-*b*-A_45_—to condense therapeutic nucleic acids. To favor the endosomal escape, an additional poly­(butyl acrylate) block was added, generating M_29_-*b*-A_29_-*b*-B_9_ and M_58_-*b*-A_52_-*b*-B_32_ triblock copolymers.

In a previous study, we showed that vaccination with these polymer/ovalbumin (pOVA) glycoplexes activated DCs in vitro and enabled the SIINFEEKL presentation via MHC class I molecules. These findings were further confirmed in vaccinated mice, where increased infiltration of both CD4^+^ and CD8^+^ T cells was observed, along with antigen-specific recognition of the SIINFEEKL OVA peptide by CD8^+^ T cells.

The primary objectives of the current study are (i) to investigate whether this platform polymeric delivery system allows coencapsulation and delivery of adjuvants—e.g., model adjuvant CpG, and enhance dendritic cell (DC) activation, and (ii) to test how the length of linear mannosylated ligands for the CD206 receptor and the presence of a hydrophobic block affect association/internalization with selected cells both in vitro and through molecular dynamic (MD) simulations.

Accordingly, these copolymers were utilized for condensing and delivering short-sequence CpG ODN as a potential cancer vaccination strategy. In these copolymers, the mannosylated block serves both to stabilize the resulting polyplexes (glycoplexes) by forming a hydrophilic corona at their surface and to act as a targeting ligand to enhance the uptake by dendritic cells (DCs). We found that mannosylated M_*x*_-*b*-A_*y*_ diblock copolymers bearing the agmatinyl cationic portion efficiently condense model ssDNA with N/P ratios in the 5–20 range, with increasing efficiency observed as the polymer length increases. Triblock copolymers M_*x*_-*b*-A_*y*_-*b*-B_*z*_ displaying an additional poly­(*n*-butyl acrylate) B_*z*_ hydrophobic block formed polyplexes at lower N/P ratios (3 for both M_29_-*b*-A_29_-*b*-B_9_, and M_58_-*b*-A_52_-*b*-B_32_). All glycoplexes were found to be biocompatible with both CD206-positive and CD206-negative cells and are stable in the presence of serum for at least 8 h. Finally, the association of glycoplexes based on both di- and triblock copolymers with CD206-expressing cells was found to be dependent on polymer composition, polymer average molar mass, and cell surface receptor density. Intracellular trafficking of M_58_-*b*-A_45_ and M_58_-*b*-A_52_-*b*-B_32_ polymer complexed with cy5-labeled model ssDNA (cy5-ssDNA) revealed slow endosomal escape and dissociation for M_58_-*b*-A_45_/cy5-ssDNA glycoplexes. Conversely, glycoplexes formed with the M_58_-*b*-A_52_-*b*-B_32_ copolymer showed fast release and nuclear localization of condensed nucleic acids. Crucially, in vitro studies showed that CpG-loaded polyplexes enhanced DC activation compared to that of free CpG. In vitro coadministration of CpG and plasmid encoding for model antigen ovalbumin (pOVA), either within the same polyplex or as separate formulations, resulted in different DC activation profiles, revealing the versatility and potential of this platform technology for the simultaneous delivery of multiple therapeutic nucleic acids in cancer vaccination.

## 2 Results and Discussion

This study aimed to evaluate mannose-targeted polymeric cations developed by our group^11^ (Figure 1A) as carriers for the active delivery of CpG ODN vaccine adjuvants to dendritic cells (Figure 1B). The block copolymers used in this work (Figure 1A) were prepared by reversible addition-fragmentation chain transfer (RAFT) polymerization and include: (i) a hydrophilic mannosylated block (M_*x*_) for selective targeting of immature DCs expressing the Mannose Receptor (MR, CD206); (ii) a positively charged agmatinyl-based block (A_*y*_), which condenses nucleic acids via its guanidinium group; and, in the case of triblock copolymers, (iii) a hydrophobic poly­(butyl acrylate) block (B_*z*_) to potentially promote endosomal escape of the nucleic acid cargo following cell uptake. Indeed, polymers bearing hydrophobic blocks have been shown to interact with lipophilic cell membranes by inducing pore formation or membrane rupture.^21,22^ More specifically, three M_*x*_-*b*-A_*y*_ diblock copolymers—M_15_-*b*-A_12_, M_29_-*b*-A_25_, and M_58_-*b*-A_45_—were synthesized with a constant M/A ∼ 1 but a different total number of repeating units, to test the effect of the polymer chain length on nucleic acid complexation and delivery. Triblock copolymers M_29_-*b*-A_29_-*b*-B_9_ and M_58_-*b*-A_52_-*b*-B_32_ were synthesized to include a poly­(butyl acrylate) block. The combined effect of these copolymer structural components is expected to enhance nucleic acid delivery by preventing extracellular or intracellular enzymatic degradation by ubiquitous or tissue-specific nucleases,^23^ and facilitating selective uptake by antigen-presenting cells through CD206-mediated endocytosis.

**Figure 1:**
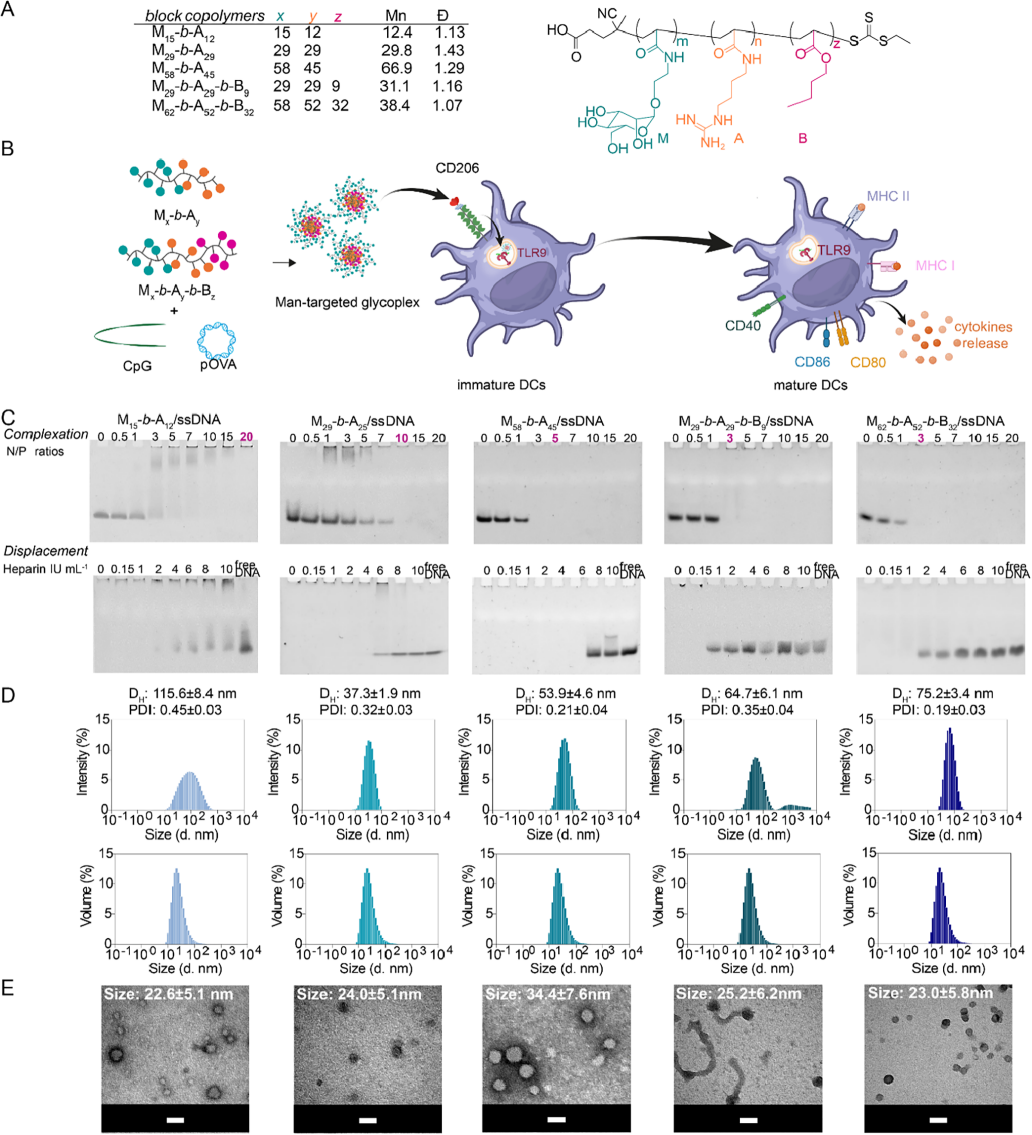
(A) Glycopolycation composition and structure. *M*_n_ and *Đ* were determined by aqueous size-exclusion chromatography (SEC) as previously reported by our group.^11^ (B) Schematic representation of glycoplex formulation and the scope of the project. M: mannopyranosyl unit; A: agmatinyl unit; B: butyl unit (modified from Bellato et al. Biomacromolecules 2022 23(12), 5148–5163.^11^ Copyright 2022 American Chemical Society). (C) ssDNA binding efficiency (complexation) of the polymers across an N/P ratio range of 1–20, along with glycoplexes stability in the presence of competing anions (heparin, 0–10 IU mL^–1^) (displacement), as determined by gel electrophoresis. Free DNA was used as a control. (D) Dynamic light scattering (DLS) analyses of polyplexes prepared in Milli-Q water at the selected N/P ratio for each polymer (in purple in the complexation gel images in (C))—and 24 μg mL^–1^ ssDNA concentration. The selected ratios for all subsequent studies are indicated in purple. DLS results are reported in intensity (top panels) and volume (bottom panels). The *D*_H_ is reported as intensity values. (E) Transmission electron microscopy (TEM) analyses of glycoplexes prepared in Milli-Q water at a polymer concentration of 0.25 mg mL^–1^. The size is reported as the average ± standard deviation (sd) of 50 polyplex measurements. Scale bar: 50 nm.

### 2.1 Glycoplexes Complexation and Stability

The ability of all copolymers to form stable glycoplexes with a 19-nucleotide (nt) long ssDNA, chosen here as a model CpG ODN analogue, was assessed via an electrophoretic mobility shift assay (EMSA) (Figure 1C, complexation). N/P ratios of 1–20 were tested to examine the effect of polymer length and composition on nucleic acid complexation. As shown in Figure 1C, the complexation efficiency of diblock copolymers increased as the polymer chain length also increased, with M_15_-*b*-A_12_, M_29_-*b*-A_25_, and M_58_-*b*-A_45_ that fully complexed ssDNA at N/P ratios of 20, 10, and 5, respectively. Notably, at the same N/P ratio, the total number of guanidine groups involved in DNA binding is identical across all polymers. However, more efficient complexation was observed for polymers with longer cationic poly­(agmatinyl acrylamide) A_*y*_ blocks, that is, when the cationic agmatinyl groups were concentrated in fewer longer chains rather than in a larger number of shorter chains.

In contrast, triblock copolymers (M_29_-*b*-A_29_-*b*-B_9_ and M_62_-*b*-A_52_-*b*-B_32_) fully complexed ssDNA at an N/P ratio of 3. This suggests that the hydrophobic poly­(butyl acrylate) block enhances nucleic acid packing, potentially via glycoplex core stabilization through hydrophobic interactions.

For subsequent studies, glycoplexes were formulated at the lowest N/P ratio for each glycopolymer that gave complete DNA complexation (Figure 1C, purple). Given that sufficient polyplex stability is crucial for the complex to reach its intended biological target, the resistance to competing anions such as those present in biological environments was tested. To this aim, heparin was utilized as a model polyanionic competitive ligand (Figure 1C, displacement). After 15 min of incubation with heparin at concentrations analogous to those found in plasma (0.1 IU mL^–1^), all glycoplexes retained their condensed ssDNA. M_29_-*b*-A_25_/ssDNA and M_58_-*b*-A_45_/ssDNA remained stable even at 4 and 6 IU mL^–1^, respectively, which are about 40–60 times higher than physiological levels, whereas M_15_-*b*-A_12_/ssDNA resisted displacement up to a heparin concentration of 1 IU mL^–1^. This showed that for polymers with an analogous mannopyranoside/agmatinyl (M/A) ratio, longer macromolecular chains complex ssDNA more efficiently—i.e., at lower N/P ratio—and form glycoplexes that are more stable in the presence of competitive polyanions, with complex stability following the trend M_58_-*b*-A_45_/ssDNA > M_29_-*b*-A_25_/ssDNA > M_15_-*b*-A_12_/ssDNA. Interestingly, despite their superior complexation efficiency at low N/P ratios, triblock copolymers formed glycoplexes that were less stable in the presence of the competing polyanion heparin (M_29_-*b*-A_29_-*b*-B_9_/ssDNA and M_62_-*b*-A_52_-*b*-B_32_/ssDNA disassembled at 1 and 2 IU mL^–1^, respectively), likely due to differences in glycoplex supramolecular organization compared to their diblock counterparts.^24^

### 2.2 Structural Characterization of Glycoplexes

Size and morphology of the polyplexes obtained by condensing 19 nt ssDNA with the library of cationic glycopolymers utilized in this work were investigated by dynamic light scattering (DLS) and transmission electron microscopy (TEM). DLS analysis (Figure 1D) confirmed that at the selected N/P ratios all polymers formed colloidal structures with relatively low polydispersity (PDI ∼ 0.2 for longer copolymers M_58_-*b*-A_45_/ssDNA and M_62_-*b*-A_52_-*b*-B_32_/ssDNA; ∼0.35 for intermediate-size copolymers M_29_-*b*-A_25_/ssDNA and M_29_-*b*-A_29_-*b*-B_9_/ssDNA; >0.4 for smaller copolymer M_15_-*b*-A_12_/ssDNA). M_15_-*b*-A_12_, M_29_-*b*-A_25_, and M_29_-*b*-A_29_-*b*-B_9_ complexes with ssDNA produced ∼40 nm particles, albeit with greater heterogeneity, while polymers with higher molar masses formed larger, more uniform particles (∼50–70 nm). Importantly, TEM images (Figure 1E) corroborated the DLS volume data, showing that high-molar-mass polymers yielded homogeneous structures, although M_62_-*b*-A_52_-*b*-B_32_/ssDNA appeared smaller (∼23 nm) than predicted by DLS (∼50 nm). The discrepancy suggests the presence of a hydrated corona in solution, which is not present in the dried samples analyzed by TEM. Additional TEM images at lower magnification are shown in Figure S1.

A unique morphology was observed for M_29_-*b*-A_29_-*b*-B_9_/ssDNA, which exhibited the coexistence of spherical (34.9 ± 6.8 nm) and rod-shaped particles (aspect ratio: 7.6; length: 192.1 ± 108.8 nm; width: 25.2 ± 6.2 nm).

### 2.3 Molecular Dynamics Simulations

To shed light on the structure of glycoplexes at a molecular level and on the copolymer–DNA interactions, we performed all-atom (AA) molecular dynamics (MD) simulations. In the simulations, 30, 8, and 2 polymer chains were taken for the M_15_-*b*-A_12_, M_29_-*b*-A_25_, and M_29_-*b*-A_29_-*b*-B_9_, respectively, as experimentally determined for full ssDNA complexation (Figure 1C). Figure 2 shows the clusters obtained at the end of MD trajectories of ssDNA in water in the presence of M_15_-*b*-A_12_, M_29_-*b*-A_25_, and M_29_-*b*-A_29_-*b*-B_9_. The clusters contain 11 copolymers in the former case and 2 copolymers in the 2 other cases, which are the lowest polymer/ssDNA ratios where complete complexation was experimentally observed. The time and length scales of AA simulations are far below those characterizing the formation of nanoparticles, with simulation running for 500 ns, while incubation times experimentally investigated for polyplex assembly generally span from 5 to 60 min;^25^ however, the simulations provide an atomic resolution view of the early stage of the polymer–DNA complexation process, which is useful to connect the experimental results on complexation (i.e., N/P ratio and stability) to the structural and chemical characteristics of the copolymers.

**Figure 2:**
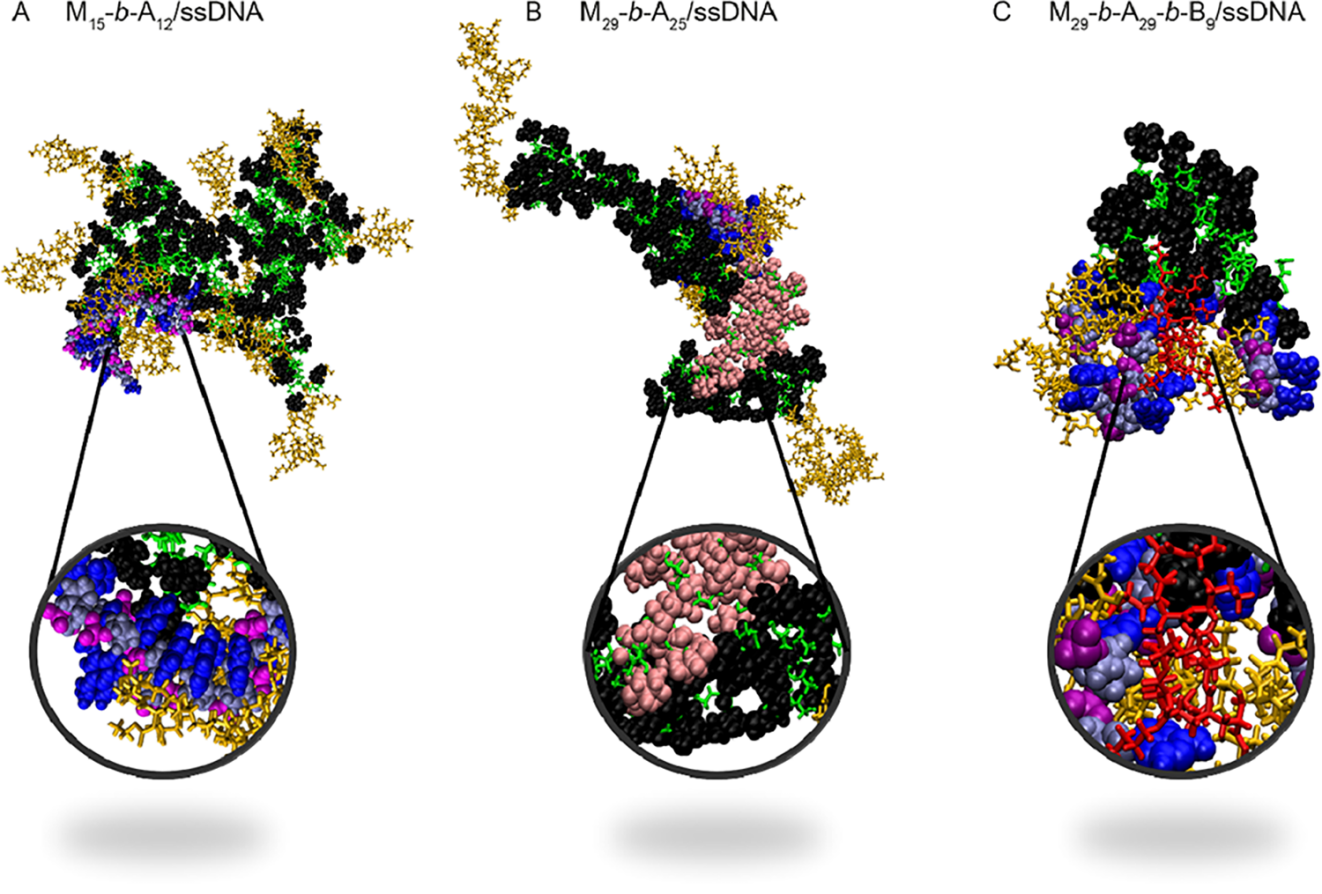
Snapshots of clusters containing one molecule of ssDNA (19-nucleotide length) and (A) 11 M_15_-*b*-A_12_ chains, (B) 2 M_29_-*b*-A_25_ chains, and (C) 2 M_29_-*b*-A_29_-*b*-B_9_ chains, at the end of MD trajectories run for 500 ns. Different colors are used for DNA (nucleobases: blue, phosphate ester groups: purple, deoxyribose: gray) and for the block copolymers (agmatinyl units A: ocher, butyl acrylate B units: red, polyacrylamide backbone of the M block: green, mannopyranosyl pendant groups: black; in (B) the mannopyranosyl pendant groups are shown either in black or pink to differentiate between mannopyranosyl groups belonging to different polymer chains).

The snapshots in Figure 2A,B show that ssDNA interacts with the cationic portions of M_15_-*b*-A_12_ and M_29_-*b*-A_25_. In both cases, the ssDNA is wrapped around the agmatinyl block, which, in the absence of DNA, would be stretched out toward water. The nucleobases contribute to the stability of the complex through base-stacking (zoomed regions in Figure 2A) and interaction with the apolar backbone and pendant groups of the glycopolymers. Interestingly, the MD simulations indicate that the mannosylated blocks tend to interact with each other, and this results in coiling of the M_*x*_ blocks and in intercopolymer attraction mediated by the mannopyranoside groups, as shown in Figure 2B. Considering that (i) the complexation via long A_*y*_ blocks is favored by entropy (a larger number of counterions and water molecules in contact with DNA and polycation are released), and (ii) the interaction of long M_*x*_ blocks can produce a denser and more compact hydrophilic corona, higher molar mass M_*x*_-*b*-A_*y*_ diblock copolymers are expected to stabilize polyplexes and to shield more effectively their core from polyanions competing with negatively charged ssDNA. Conversely, in glycoplexes formed by shorter M_*x*_-*b*-A_*y*_ diblock copolymers, this entanglement between M_*x*_ blocks of different polymer chains was not observed. This lack of entanglement may make the mannopyranoside groups at the surface of glycoplexes more accessible for lectin binding. This may explain the more selective association of glycoplexes formed by diblock copolymers with shorter mannosylated M_*x*_ to CD206^+^ cells, which we observed in subsequent cell uptake experiments (vide infra).

In the case of M_*x*_-*b*-A_*y*_-*b*-B_*z*_ triblock copolymers (MD in Figure S2), MD simulations show interaction of hydrophobic poly­(butyl acrylate) blocks with the nucleobases (Figure 2C). This leads to the formation of compact structures where ssDNA is in contact with both the cationic (A_*y*_) and the hydrophobic (B_*z*_) blocks of copolymers, which may explain why these M_*x*_-*b*-A_*y*_-*b*-B_*z*_ materials showed complete ssDNA complexation at low N/P ratios (Figure 1C). In a previous study,^26^ coarse-grained MD simulations indicated that polyplexes formed from triblock copolymers containing hydrophilic, hydrophobic, and cationic blocks possess hydrophobic patches on their surface, and a proportion of nucleobases is weakly bound to these hydrophobic domains rather than to the positively charged repeating units of the triblock copolymer. Thus, these nucleobases could be more prone to interacting with competitive ionic ligands, which would explain the relatively low stability of M_*x*_-*b*-A_*y*_-*b*-B_*z*_/ssDNA glycoplexes in the presence of the competing polyanion heparin, observed in our study (Figure 1C).

### 2.4 Surface Charge and Colloidal Stability of M_*x*_-*b*-A_*y*_/ssDNA and M_*x*_-*b*-A_*y*_-*b*-B_*z*_/ssDNA Glycoplexes

The zeta potential (ζ) of colloidal systems affects not only their stability, but also their uptake by cells, their biocompatibility, and—for polyplexes—their transfection efficacy.^27,28^ Polyplexes are often formulated at N/P ratios above one, which leads to nanoparticles with a slightly positive ζ potential. On one hand, this results in electrostatic repulsion among positively charged particles, which reduces particle aggregation; on the other hand, as the surface of cell plasma membranes is slightly anionic, positive charges on polyplexes can cause nonspecific binding and uptake by cells, which might reduce selective targeting of specific cells.^29^

Before ssDNA binding, all polymers are expected to be positively charged due to guanidinium groups. Upon ssDNA complexation, positive ζ potential values result from an excess of positive charges (i.e., N vs P value) applied during glycoplex formulation. As shown in Figure 3A, among all glycoplexes, those based on triblock copolymers were characterized by lower ζ potential values (M_29_-*b*-A_29_-*b*-B_9_/ssDNA: 0.88 mV; M_62_-*b*-A_52_-*b*-B_32_/ssDNA: 0.10 mV), in agreement with their low N/P ratio requirements. The positive ζ potential for M_15_-*b*-A_12_/ssDNA glycoplexes was consistent with the excess of positively charged nitrogen atoms (N) in the agmatinyl units used to fully condense ssDNA (N/P ratio: 20). In general, despite near-neutral ζ potential values, glycoplexes exhibited high colloidal stability (Figure 3A).

**Figure 3:**
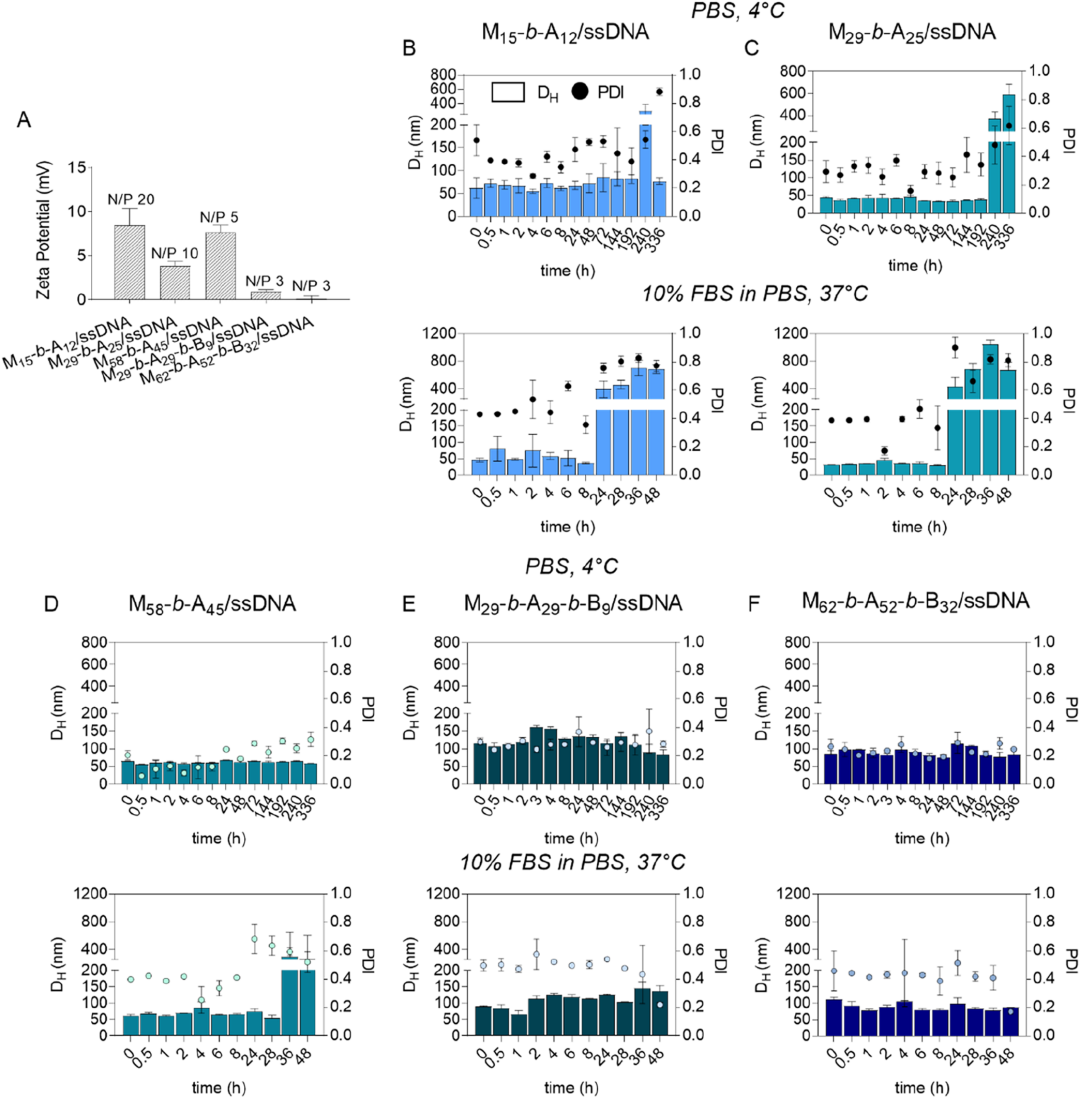
(A) ζ potential values for ssDNA-loaded glycoplexes prepared in 5 mM *N*-(2-hydroxyethyl)­piperazine-*N*′-ethanesulfonic acid (HEPES) at a polymer concentration of 0.1 mg mL^–1^. Glycoplexes were formulated at the N/P ratios selected by complexation studies: M_15_-*b*-A_12_/ssDNA N/P ratio 20, M_29_-*b*-A_25_/ssDNA N/P ratio 10, M_58_-*b*-A_45_/ssDNA N/P ratio 5, M_29_-*b*-A_29_-*b*-B_9_/ssDNA, and M_62_-*b*-A_52_-*b*-B_32_/ssDNA N/P ratio 3. (B–F) Stability profile of glycoplexes stored in phosphate-buffered saline (PBS) at 4 °C for 10 days (top panels) or in PBS supplemented with 10% fetal bovine serum (FBS) at 37 °C for 48 h (bottom panels) as assessed by DLS (*D*_H_ are reported as intensity-weighted percentages). M_15_-*b*-A_12_/ssDNA at the N/P ratio of 20 (B), M_29_-*b*-A_25_/ssDNA at the N/P ratio of 10 (C), M_58_-*b*-A_45_/ssDNA at the N/P ratio of 5 (D), M_29_-*b*-A_29_-*b*-B_9_/ssDNA at the N/P ratio of 3 (E), and M_62_-*b*-A_52_-*b*-B_32_/ssDNA at the N/P ratio of 3 (F).

Long-term storage stability of glycoplexes was assessed in PBS at 4 °C, monitoring changes in hydrodynamic diameter (*D*_H_) by DLS over 2 weeks (Figure 3B–F, top panels). All complexes remained stable for at least 8 days, with M_58_-*b*-A_45_/ssDNA, M_29_-*b*-A_29_-*b*-B_9_/ssDNA, and M_62_-*b*-A_52_-*b*-B_32_/ssDNA retaining their initial size throughout. A minor fraction of aggregates (comprising <5% of the population by size) appeared in M_58_-*b*-A_45_/ssDNA after 24 h, as also confirmed by increased PDI values. Interestingly, ssDNA complexes with the triblock copolymers M_29_-*b*-A_29_-*b*-B_9_ exhibited enhanced stability compared to ssDNA complexes with the diblock counterpart M_29_-*b*-A_25_.

When incubated in the presence of serum at 37 °C (Figure 3B–F, bottom panels), M_29_-*b*-A_29_-*b*-B_9_/ssDNA and M_62_-*b*-A_52_-*b*-B_32_/ssDNA remained stable for 48 h, while M_58_-*b*-A_45_/ssDNA retained stability for 28 h. In contrast, M_15_-*b*-A_12_/ssDNA and M_29_-*b*-A_25_/ssDNA showed aggregation at 24 h, highlighting the superior serum stability of ssDNA complexes with M_29_-*b*-A_29_-*b*-B_9_ and M_62_-*b*-A_52_-*b*-B_32_ triblock copolymers.

While at this stage the reasons for the lower stability of M_15_-*b*-A_12_/ssDNA and M_29_-*b*-A_25_/ssDNA complexes under storage conditions were not investigated in detail, it is possible that, in the absence of a poly­(butyl acrylate) (B_*z*_) hydrophobic block, shorter polymers form less compact structures, making them more susceptible to aggregation over time compared to glycoplexes formed from longer polymers.

These experiments suggest that the enhanced storage stability of glycoplexes based on M_*x*_-*b*-A_*y*_-*b*-B_*z*_ triblock copolymers may be attributed to additional interchain hydrophobic interactions between poly­(butyl acrylate) B_*z*_ blocks, interactions between the poly­(butyl acrylate) B_*z*_ chains and the nucleobases, or a combination of both. This effect was particularly pronounced for glycoplexes formed with M_62_-*b*-A_52_-*b*-B_32_, which possess a longer poly­(butyl acrylate) block and maintained almost the initial size all the duration of the study. In contrast, a moderate increase in hydrodynamic diameter (*D*_H_), from 90.1 ± 2.37 nm at time 0 to 113.0 ± 9.12 nm at time 2 h, was observed for M_29_-*b*-A_29_-*b*-B_9_/ssDNA glycoplexes incubated in PBS supplemented with 10% fetal bovine serum (FBS) (Figure 3B–F). Conversely, in polyplexes based on M_*x*_-*b*-A_*y*_ diblock copolymers, a marked increase in size was observed after 24 or 36 h, which could be ascribed to protein adsorption upon serum exposure. This adsorption over time may be driven by electrostatic interactions due to the positive net charge of the polyplexes formed with M_*x*_-*b*-A_*y*_ as compared to that obtained with the M_*x*_-*b*-A_*y*_-*b*-B_*z*_ triblock copolymers that would favor interaction with negatively charged protein, such as albumin. However, further studies are required to confirm this hypothesis.

Overall, storage stability experiments indicated higher stability under physiological-mimicking conditions and in the presence of serum proteins for ssDNA glycoplexes based on M_*x*_-*b*-A_*y*_-*b*-B_*z*_ triblock copolymers. These complexes, however, showed a higher tendency to disassemble in the presence of the competing polyanionic ligand heparin, compared to polyplexes based on M_*x*_-*b*-A_*y*_ diblock copolymers.

### 2.5 Biocompatibility and Hemolytic Activity of M_*x*_-*b*-A_*y*_ and M_*x*_-*b*-A_*y*_-*b*-B_*z*_ Copolymers and Their Corresponding ssDNA Glycoplexes

The biocompatibility of glycoplexes was assessed with Chinese hamster ovary cell lines, both wild type (CHO) and stably transfected to express the mannose receptor (CHO-CD206^+^), following 24 h of incubation and evaluation via the 3-(4,5-dimethylthiazol-2-yl)-2,5-diphenyl tetrazolinium bromide (MTT) viability assay. CHO-CD206^+^ cells were included to assess the impact of CD206-mediated glycoplex internalization on the cell viability. The results (Figure S3) demonstrated excellent biocompatibilities for all glycoplexes in the 0–1 μM ssDNA concentration range, which includes the 125 nM ssDNA concentration used in the subsequent uptake studies. No significant differences were observed between the two cell lines despite their markedly different glycoplex internalization profiles (Figure S4), with cell viability exceeding 80% for nearly all formulations, even at the highest concentrations tested.

Efficient endosomal release and appropriate intracellular trafficking (i.e., reaching the nucleus or the cytoplasm, depending on the site of action) remain major challenges in nonviral nucleic acid delivery. The ability of drug delivery systems to escape from endolysosomal compartments is crucial to prevent degradation of nucleic acid therapeutics by acidic pH and lysosomal nucleases. To address this challenge, delivery systems have been engineered to escape this intracellular barrier.^30,31^ In this study, M_*x*_-*b*-A_*y*_-*b*-B_*z*_ copolymers, possessing poly­(butyl acrylate) hydrophobic blocks, were tested based on previous findings showing that hydrophobic moieties in nucleic acid polyplexes can disrupt endosomal phospholipid membranes and promote escape.^1,32,33^

To evaluate the membrane-disruptive potential of polymers and glycoplexes, these materials were incubated with rat red blood cells (RBCs) at polymer concentrations ranging from 0.005 to 1 mg mL^–1^. The extent of RBC membrane destabilization, leading to hemoglobin release, is often utilized as an indicator of endosomal escape potential.^34^ The assay was performed under both physiological (pH 7.4) and early endosome-mimicking (pH 6.5) conditions (Figure 4).

**Figure 4:**
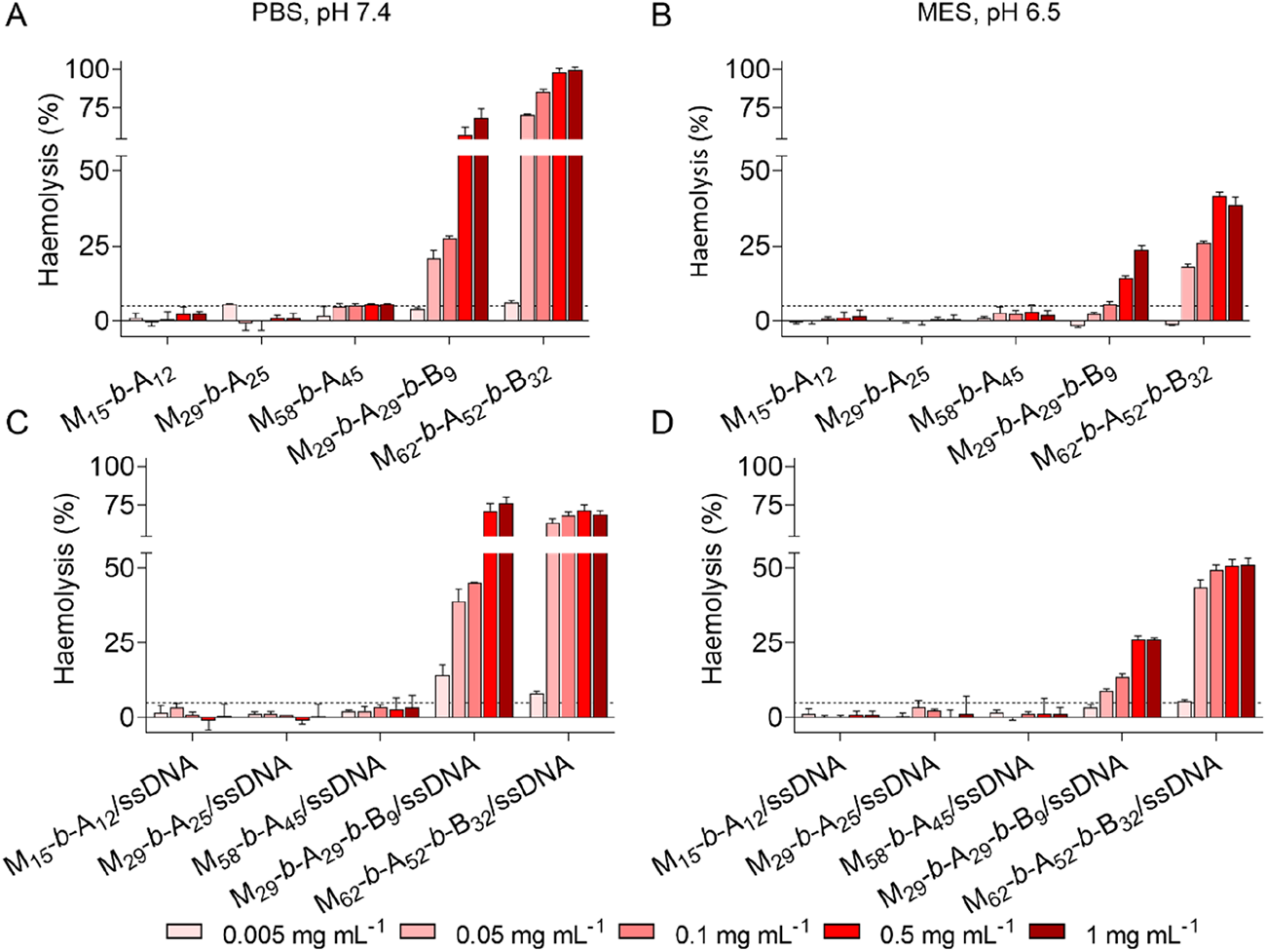
Hemolysis assay of glycopolymers and glycopolymer/ssDNA glycoplexes. Relative percentage of rat RBCs lyses upon incubation with free polymers (A, B) or glycoplexes (C, D) in the 0.005–1 mg mL^–1^ polymer concentration range performed in (A, C) PBS, pH 7.4, and (B, D) 10 mM 2-(*N*-morpholino)­ethanesulfonic acid (MES), 149 mM NaCl, pH 6.5 (MES, pH 6.5). Samples incubated with buffer only or buffer containing 1% Triton X-100 were used to normalize data as negative (0% lysis) and positive (100% lysis) controls, respectively. Dashed lines indicate the 5% hemolysis threshold.

Hemolytic activity of free M_*x*_-*b*-A_*y*_ and M_*x*_-*b*-A_*y*_-*b*-B_*z*_ polymers generally increased with polymer concentration and chain length, with a more pronounced effect observed for polymers containing poly­(butyl acrylate) blocks, as anticipated. In particular, M_62_-*b*-A_52_-*b*-B_32_ exhibited the highest hemolytic activity (>99% and 38% RBC lysis at 1 mg mL^–1^ under pH 7.4 and 6.5, respectively), consistent with its longer hydrophobic block compared to the other amphiphilic triblock copolymer tested, M_29_-*b*-A_29_-*b*-B_9_. In contrast, diblock copolymers exhibited minimal hemolytic activity (<5% for M_15_-*b*-A_12_, M_29_-*b*-A_25_, and M_58_-*b*-A_45_) even at the highest tested concentrations and at both pH values, supporting the good biocompatibility observed in cell viability assays.

A similar trend was observed for glycoplexes, with M_*x*_-*b*-A_*y*_-based polyplexes exhibiting hemolytic activity below 5%, while M_*x*_-*b*-A_*y*_-*b*-B_*z*_-based glycoplexes showed hemolysis levels comparable to those of their corresponding free polymer. This suggests that after ssDNA complexation, the poly­(butyl acrylate) block in M_*x*_-*b*-A_*y*_-*b*-B_*z*_ remains accessible for interaction with cellular membranes. This observation aligns with molecular dynamics simulations illustrated in Figure 2C, where red patches representing the poly­(butyl acrylate) block are exposed on the complex outer surface. Interestingly, the hemolytic activity of these materials was found to be higher at pH 7.4 than at pH 6.5. While further ad hoc investigations are needed to elucidate this behavior, it may reflect greater exposure of the poly­(butyl acrylate) hydrophobic blocks under neutral conditions, which in turn translates into higher hemolytic activity. Despite a certain extent of hemolytic activity observed with butyl acrylate-containing copolymers and glycoplexes, these materials exhibited excellent biocompatibility in cellular environments, as shown by our MTT assay results. Moreover, their intended application as vaccine carriers involves subcutaneous administration, thereby minimizing direct contact with red blood cells and plasma proteins. Nonetheless, in our first work, we demonstrated the stability of glycoplexes formed with M_*x*_-*b*-A_*y*_ and M_*x*_-*b*-A_*y*_-*b*-B_*z*_ copolymers, for at least 24 h when incubated in the presence of serum protein. Moreover, M_58_-*b*-A_45_ effectively protected nucleic acid from degradation for a minimum of 24 h, while M_62_-*b*-A_52_-*b*-B_32_ shows marginal degradation at 6 h.

### 2.6 Polymer/ssDNA Glycoplexes Association to Cells Expressing CD206 and Intracellular Trafficking

In this study, we aimed to evaluate the selective binding of polyplexes to CD206-expressing cells and their subsequent receptor-mediated internalization. The selective association of the targeted glycoplexes by CD206-expressing cells was assessed by flow cytometry (FC) using cyanine-3 (cy3)-labeled ssDNA (ssDNA-cy3) as a model nucleic acid cargo. Wild-type CHO cells, which do not express the CD206 receptor, were used as a negative control, while CHO-CD206^+^ cells served as the experimental group. As suggested previously,^35^ to investigate cell binding events mediated by CD206, CHO-CD206^+^ cells are better suited than primary CD206-expressing immune cells, where this receptor may not be expressed at a stable level across all cells, and where variation in CD206 expression can be dependent on cell activation states. Moreover, such immune cells may also express other lectin receptors, such as DC-SIGN and Langerin, which may complicate the interpretation of binding events. Finally, wild-type CHO cells serve as ideal negative controls (nonexpressing cells), as they are identical to CHO-CD206^+^ cells, the experimental group, except for the lack of CD206 expression. Glycoplexes uptake by DC2.4 murine immortalized dendritic cells, which more closely resemble the phenotype of the target immune cells, was then also investigated. Indeed, DC2.4 cell line has been extensively utilized as a model in vaccination assays due to its ability to effectively recapitulate both antigen uptake and dendritic cell maturation processes.^36^ Accordingly, glycoplexes were formulated at a 125 nM concentration of ssDNA-cy3, at which no significant toxicity was observed in our previous biocompatibility experiments (Figure S3). Diblock copolymers-based glycoplexes, particularly M_15_-*b*-A_12_/cy3-ssDNA and M_29_-*b*-A_25_/cy3-ssDNA, exhibited higher selectivity for CHO-CD206^+^ cells, with 8.9 and 4.5-fold greater internalization, respectively, compared to CHO cells (Figure 5A,B, 30 min incubation). This selectivity inversely correlated with the length of the diblock copolymer, as M_58_-*b*-A_45_/cy3-ssDNA copolymer displayed 2.5-fold higher association with CD206-expressing cells. The presence of the B_*z*_ block abolished this selectivity, as no statistically significant difference in association was observed between CHO and CHO-CD206^+^ cell lines for M_29_-*b*-A_29_-*b*-B_9_/cy3-ssDNA and M_62_-*b*-A_52_-*b*-B_32_/cy3-ssDNA. This finding was unexpected, as M_15_-*b*-A_12_/cy3-ssDNA and M_29_-*b*-A_25_/cy3-ssDNA glycoplexes were assembled at much higher N/P ratios than M_58_-*b*-A_45_/cy3-ssDNA, for which an excess of positive charges was anticipated to promote nonspecific uptake. However, the results indicated that shorter polymers exhibited greater selectivity, suggesting that shorter mannosyl chains might adopt a spatial arrangement that enhances the interaction with the C-type lectin-like domain of CD206, corroborating the results obtained with MD simulations showing that long mannosylated blocks tend to interact with each other rather than being exposed to the solvent. On the other hand, it must also be considered that glycoplexes characterized by higher N/P values also involve a higher number of polymer chains. Consequently, there is an average of ∼475, 220, and 122 mannopyranosyl sugar units per oligonucleotide for M_15_-*b*-A_12_/ssDNA, M_29_-*b*-A_25_/ssDNA, and M_58_-*b*-A_45_/ssDNA, respectively. Thus, an increase in the number of mannopyranosyl units displayed on each complex is associated with greater selectivity toward CD206-expressing cells. Additionally, results from the hemolysis assay and MD simulations suggest that the poly­(butyl acrylate) blocks of M_29_-*b*-A_29_-*b*-B_9_ and M_62_-*b*-A_52_-*b*-B_32_ have access to the glycoplexes outer surface, promoting noncell-specific interactions with cell membranes. The efficient uptake mediated by the poly­(butyl acrylate) block in CHO and CHO-CD206^+^ cells persisted after 1 h incubation (Figure S4), with prolonged exposure leading to significantly higher internalization of triblock-based glycoplexes.^37^

**Figure 5:**
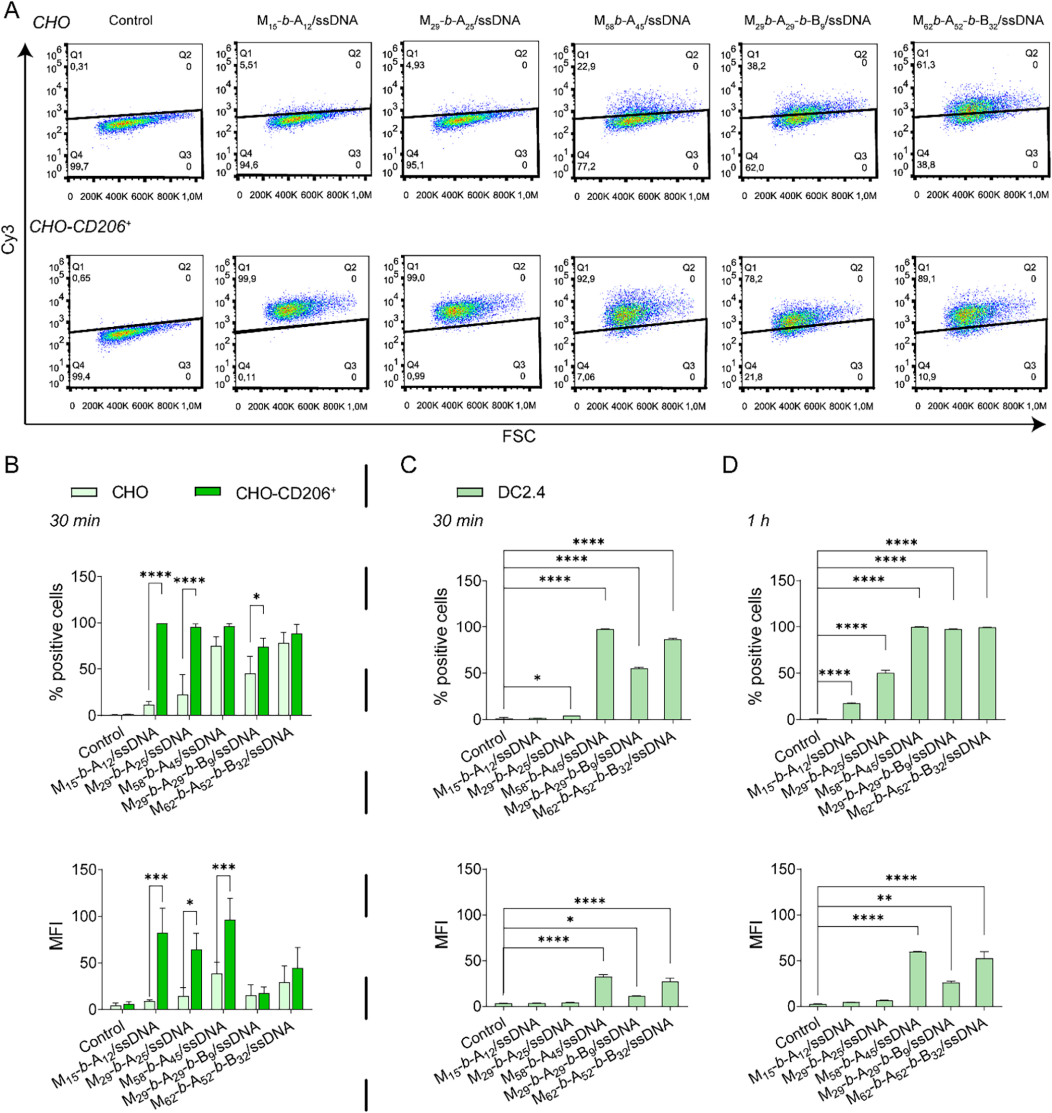
Association of M_*x*_-*b*-A_*y*_- and M_*x*_-*b*-A_*y*_-*b*-B_*z*_-based glycoplexes with CHO, CHO-CD206^+^ and DC2.4, as assessed by flow cytometry. (A) Representative dot plots of cy3 fluorescence intensity versus forward scatter (FSC), of CHO (top panels) and CHO-CD206^+^ (bottom panels) cell lines after treatment with copolymer/cy3-ssDNA glycoplexes. (B–D) Mean fluorescence intensity (MFI) values and percentage of positive cells for CHO (green box solid) and CHO-CD206^+^ (green box solid) cells after 30 min incubation, and for (C, D) DC2.4 (green box solid) cells after 30 min and 1 h incubation with 125 nM cy-3 labeled M_15_-*b*-A_12_/cy3-ssDNA at the N/P of 20, M_29_-*b*-A_25_/cy3-ssDNA at the N/P of 10, M_58_-*b*-A_45_/cy3-ssDNA at the N/P of 5, and M_29_-*b*-A_29_-*b*-B_9_/cy3-ssDNA and M_62_-*b*-A_52_-*b*-B_32_/cy3-ssDNA both at the N/P of 3. Data are presented as means ± sd (*n* = 3, *p < 0.05, **p < 0.01, ***p < 0.001, ****p < 0.0001).

However, when the same assay was performed with DC2.4 murine dendritic cells (Figure 5C,D), the association of M_15_-*b*-A_12_/cy3-ssDNA and M_29_-*b*-A_25_/cy3-ssDNA was negligible compared to the other formulations at both 30 min and 1 h incubation times. This could be explained by the lower surface expression of CD206 in DC2.4 cells compared to CHO-CD206^+^ cells (Figure S5), hence suggesting that the receptor density on the cell plasma membrane plays a role in internalization efficiency and that the latter depends on the length of the mannosylated M_*x*_ block. Accordingly, longer polymer chains appeared to induce higher cell uptake under conditions of reduced CD206 expression, as in DC2.4 cells, where multivalent interactions with multiple mannose receptors could enhance CD206-mediated cell association of glycoplexes.^6^ After 1 h incubation, transfection efficiency in DC2.4 cells was 17.34% for M_15_-*b*-A_12_/cy3-ssDNA and 50.08% for M_29_-*b*-A_25_/cy3-ssDNA, while all other glycoplexes achieved nearly 100%. Overall, M_58_-*b*-A_45_/ssDNA glycoplex demonstrated the highest association with CD206-expressing cells compared to all other formulations, while maintaining good selectivity for CD206^+^ cells. Consequently, M_58_-*b*-A_45_ and M_62_-*b*-A_52_-*b*-B_32_ were found to be the most promising materials in this context. Glycoplexes formulated with M_58_-*b*-A_45_ showed a good level of selectivity for CD206-expressing cells, whereas those formulated with M_62_-*b*-A_52_-*b*-B_32_ showed high but nonspecific cell association. Thus, glycoplexes assembled from these two glycopolymers were selected for subsequent studies in this work. Importantly, the same glycopolymers were previously shown to efficiently transfect DC2.4 dendritic cells using a plasmid encoding enhanced green fluorescence protein (pEGFP), achieving transfection efficiencies of 5 and 17% for M_58_-*b*-A_45_- and M_62_-*b*-A_52_-*b*-B_32_-based glycoplexes, respectively.^11^

Following cell uptake of polymer/nucleic acid glycoplexes, efficient endosomal escape is necessary to prevent nucleic acid degradation by lysosomal nucleases^38^ and ensure proper intracellular trafficking of therapeutic nucleic acids to the cytoplasm or nucleus, where they can exert their intended biological effect. Here, the internalization and intracellular localization of M_58_-*b*-A_45_/cy5-ssDNA and M_62_-*b*-A_52_-*b*-B_32_/cy5-ssDNA in DC2.4 cells were investigated by confocal scanning laser microscopy.

Cells were incubated with free cy5-ssDNA or glycoplexes for 15 min (Figure S6A) or 30 min to allow internalization (Figures 6A and S6B) before staining with endosomal and lysosomal markers early endosome antigen 1 (EEA-1) and lysosomal-associated membrane protein 1 (LAMP-1), respectively, to track intracellular trafficking of ssDNA. Confocal images were acquired immediately after incubation. In another set of experiments, cells were treated with free cy5-ssDNA or glycoplexes for 30 min followed by a 2 h chase period in fresh medium (Figures 6B and S6C), to monitor endosomal processing of glycoplexes and their components over time.

**Figure 6:**
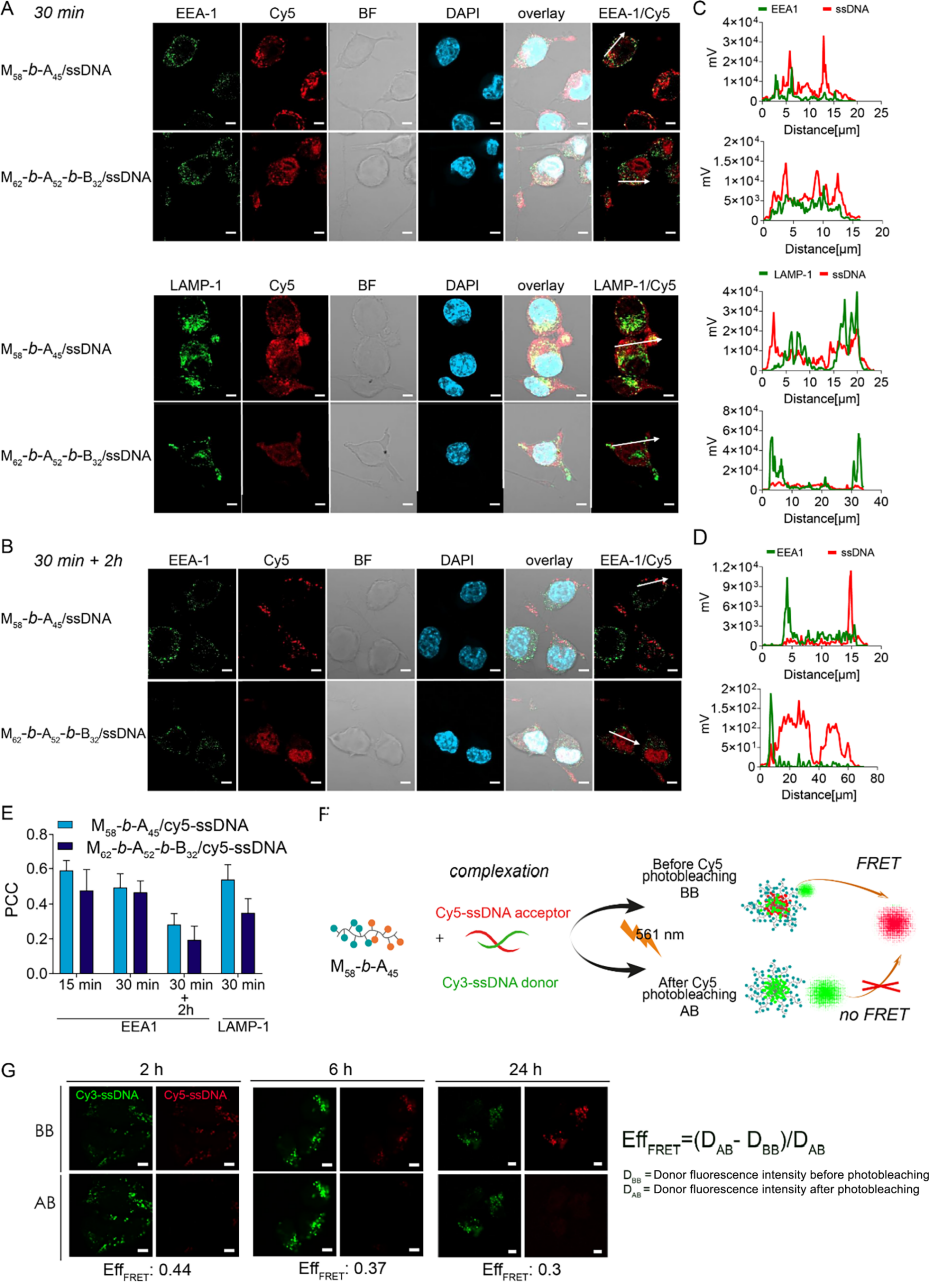
(A, B) Intracellular trafficking of glycoplexes in DC2.4 cells. Confocal microscopy images of DC2.4 cells incubated for 30 min with M_58_-*b*-A_45_/cy5-ssDNA and M_62_-*b*-A_52_-*b*-B_32_/cy5-ssDNA were stained in green for early endosomes (EEA-1) or lysosomes (LAMP-1). (A) Images acquired immediately after incubation, or (B) 2 h post-incubation. Scale bar: 5 μm. (C, D) Normalized fluorescence intensity line scans for EEA-1 or LAMP-1 (green) and cy5-labeled glycoplexes (red) channels across cell areas indicated by white arrows in the overlay images. (E) Pearson correlation coefficients (PCC) were used to estimate colocalization of cy5-ssDNA at different time points: with early endosomes at 15 and 30 min incubation, or at 30 min incubation followed by 2 h post-incubation, and with lysosomes after 30 min incubation. Pearson correlation coefficients (PCC) values represent averages from analysis of at least 10 cells across 3 different fields. Statistical significance was determined by one-way analysis of variance (ANOVA). Error bars indicate sd. (F) Schematic representation of the Förster Resonance Energy Transfer (FRET) experiment with glycoplexes formulated with a mix of cy3-ssDNA (donor) and cy5-ssDNA (acceptor), showing recorded emission before (BB) and after (AB) acceptor photobleaching. (G) Energy transfer efficiency values at 2, 6, and 24 h incubation are shown below the confocal images acquired before (BB, panels above) and after (AB, panels below) acceptor photobleaching. FRET was calculated as the energy transfer efficiency (Eff_FRET_).

M_58_-*b*-A_45_/cy5-ssDNA glycoplexes showed higher colocalization with both the endosomal marker EEA-1 and lysosomal marker LAMP-1 compared to M_62_-*b*-A_52_-*b*-B_32_/cy5-ssDNA, as shown by normalized fluorescence intensity line scans for EEA-1 or LAMP-1 (green) and cy5-labeled glycoplexes (red) (Figure 6C,D), as well as by the calculated Pearson correlation coefficients (Figure 6E). This suggests that M_62_-*b*-A_52_-*b*-B_32_/cy5-ssDNA undergoes more rapid endosomal escape, likely due to the interaction of its poly­(butyl acrylate) B_*z*_ blocks with cell membranes, consistent with our hemolytic assay data. Indeed, cy5-ssDNA from the M_62_-*b*-A_52_-*b*-B_32_/cy5-ssDNA glycoplex was found to quickly migrate from endosomes to the cytoplasm and ultimately to the nucleus (Figure 6B). In contrast, cy5-ssDNA from M_58_-*b*-A_45_/cy5-ssDNA exhibited a slower release profile, as indicated by the higher colocalization with EEA-1 and LAMP-1 compared to M_62_-*b*-A_52_-*b*-B_32_/cy5-ssDNA. After 30 min of incubation followed by a 2 h chase period, both polyplexes showed decreased colocalization with the endosomal marker EEA-1, supporting their endosomal escape.

The fate of the glycoplexes following cell internalization—i.e., whether/when glycoplexes disassemble into their copolymer and ssDNA components—was investigated by fluorescence resonance energy transfer (FRET) experiments, following a previously described approach.^39–41^ Donor dye cy3-ssDNA and acceptor dye cy5-ssDNA were coloaded into the same M_58_-*b*-A_45_/ssDNA glycoplex to monitor the energy transfer from cy3 to cy5, serving as an indicator of glycoplex integrity (Figure 6F). Accordingly, intracellular disassembly of glycoplexes would result in spatial separation of cy3-ssDNA donor dye and cy5-ssDNA acceptor dye, causing loss of FRET signal. Conversely, the presence of a FRET signal indicates that the two probes remain colocalized within polyplexes.^41^ At this stage, photobleaching of the cy5 fluorophore acceptor at λ = 561 nm results in increased cy3 donor fluorescence, further confirming the initial presence of an FRET effect.

Here, CHO-CD206^+^ cells were pulsed for 2 h with M_58_-*b*-A_45_/cy3-ssDNA-cy5-ssDNA and then chased for 2, 6, or 24 h. Cy3-ssDNA and cy5-ssDNA emissions before (BB) and after (AB) acceptor (cy5) photobleaching were recorded at each time point (Figure 6G). The increase in cy3 emission after cy5 bleaching, due to a decrease of Förster Resonance Energy Transfer (FRET), was used to calculate the energy transfer efficiency (Eff_FRET_),^42^ as illustrated in Figure 6G. The Eff_FRET_ values decreased from 0.44 to 0.3 over time, confirming that a proportion of glycoplexes disassemble, releasing their ssDNA cargo. Combined with Pearson correlation coefficient values (Figure 6E), which indicate only moderate colocalization with both early endosomes and lysosomes, these findings suggest that M_58_-*b*-A_45_/ssDNA and M_62_-*b*-A_52_-*b*-B_32_/ssDNA glycoplexes can escape endosomes and disassemble to release their cargo.

### 2.7 DCs Activation by CpG-Loaded Glycoplexes

The effect of the CpG adjuvant delivered through M_58_-*b*-A_45_ and M_62_-*b*-A_52_-*b*-B_32_ glycoplexes on activation of JAWSII dendritic cells (DCs) was then tested. JAWSII cells exhibit an immature DC phenotype (Figure S7),^43^ and thus offer a suitable model to test adjuvants focusing on the initial stage of DCs maturation and activation,^44,45^ rather than CD8^+^ and CD4^+^ T cell response. As the CpG adjuvant, class A CpG ODN 1585, possessing a phosphodiester backbone and palindromic CpG motifs at the center of its sequence,^18^ was chosen because it is a potent TLR9 agonist of this class.

Initially, glycoplexes were assembled from CpG and either M_58_-*b*-A_45_ or M_62_-*b*-A_52_-*b*-B_32_. Both polymers efficiently complexed the short DNA sequence of CpG at an N/P ratio of 3 (Figure S8A,B). Similar sizes were observed by TEM, as shown in the images reported in Figure S8C, with 27.4 ± 7.1 and 29.4 ± 7.3 nm for M_58_-*b*-A_45_/CpG and M_62_-*b*-A_52_-*b*-B_32_/CpG glycoplexes, respectively.

Next, JAWSII cells were treated with copolymer/CpG glycoplexes or with CpG alone for 24 h at a CpG concentration of 1.375 μg mL^–1^, followed by analysis of surface expression of DC activation markers (Figure 7A–C)—i.e., MHC class II (MHC II), which is crucial for antigen presentation and T-cell activation initiating the adaptive immune response,^46^ as well as the costimulatory molecules CD40, CD80 and CD86, which interact with CD28 and CD40L on T cells to stimulate their full activation.^47,48^ Upregulation of expression of costimulatory molecules and antigen-presenting MHC molecules enables DCs to elicit adaptive immunity, thus playing a crucial role in DC maturation and vaccination.^49,50^

**Figure 7:**
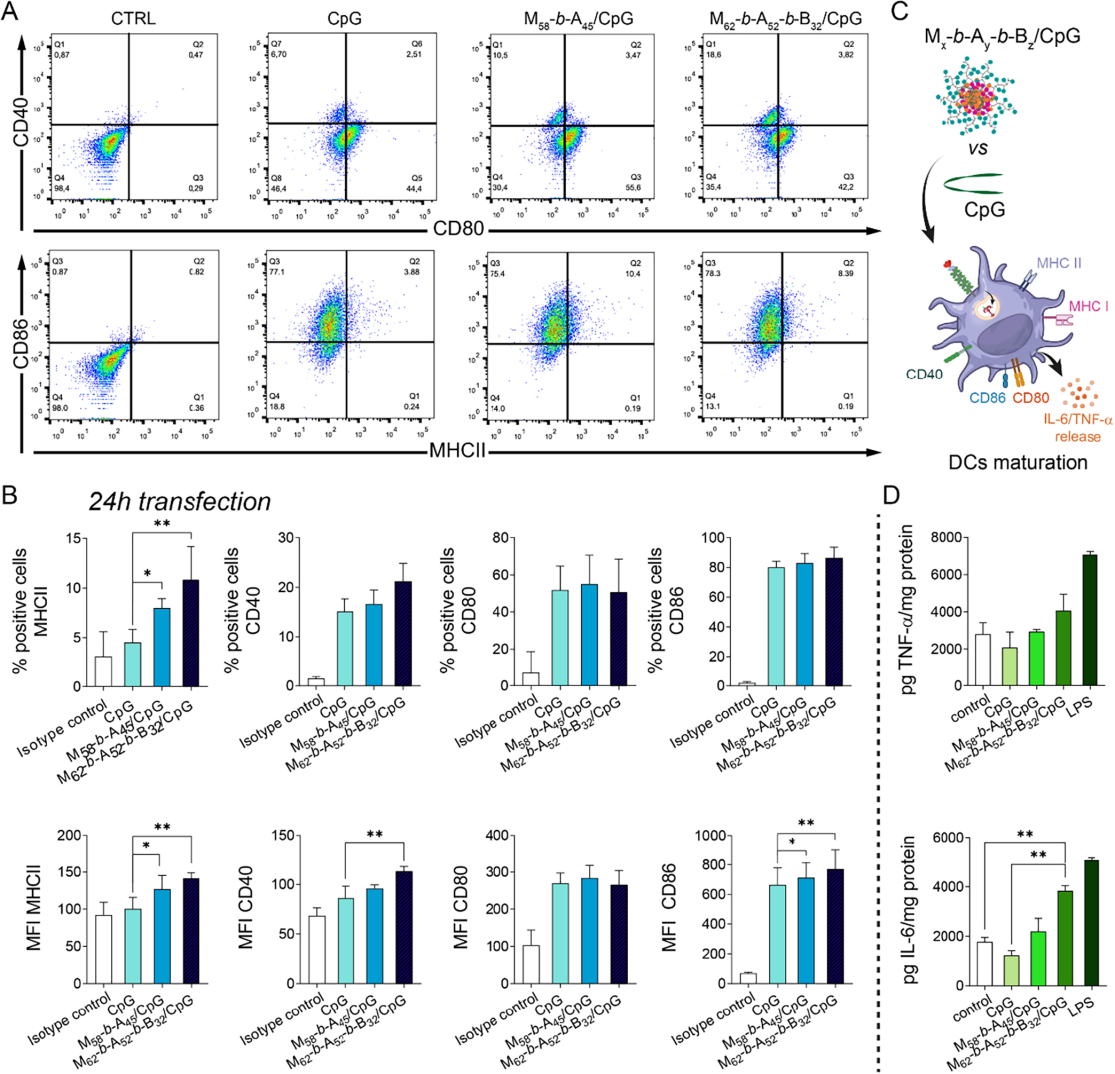
JAWSII dendritic cell activation induced by polymer/CpG glycoplexes. (A) Dot plot showing flow cytometry (FC) analysis and (B) expression levels of MHC class II, CD40, CD80, and CD86 reported as % positive cells (top panels) and as mean fluorescence intensity (MFI, bottom panels) of JAWSII DCs (labeled with antibodies CD40-APC/CD80-FITC or MHC class II-APC/CD86-PE) after incubation for 24 h (24 h transfection) with CpG alone, or M_58_-*b*-A_45_/CpG and M_62_-*b*-A_52_-*b*-B_32_/CpG glycoplexes. Untreated cells (control) and CpG alone were used as controls. Data are reported as average ± sd of three independent experiments performed in triplicate. (C) Schematic representation of CpG ODN 1585 immunostimulation mediated via Toll-like receptor 9 (TLR9) activation in the endosomes. (D) Tumor necrosis factor-α (TNF-α) and IL-6 cytokines release by JAWSII cells induced 24 h transfection with polymers/CpG polyplexes. Untreated cells (control), CpG, and lipopolysaccharide (LPS) alone were used as controls. Data are reported as average ± sd of three independent experiments performed in triplicate (*n* = 3, *p < 0.05, **p < 0.01).

Both M_58_-*b*-A_45_/CpG and M_62_-*b*-A_52_-*b*-B_32_/CpG significantly up-regulated the expression of MHC II and CD86, relative to CpG alone, with M_62_-*b*-A_52_-*b*-B_32_/CpG also inducing upregulation of CD40 (Figure 7A,B). Importantly, M_58_-*b*-A_45_/CpG and M_62_-*b*-A_52_-*b*-B_32_/CpG induced TNF-α and IL-6 secretion, as assessed by enzyme-linked immunosorbent assay (ELISA), whereas CpG alone had no detectable effect (Figures 7D and S9). It is well known that the activation of TLR9 by CpG motifs in DCs triggers the production of pro-inflammatory cytokines such as TNF-α and IL-6 that play key roles in immunity.^51^ Thus, altered secretion of these pleiotropic cytokines confirmed effective delivery of CpG to JAWSII DCs. As previously reported,^52^ cytokine levels induced by polymer/CpG complexes were lower than those observed in LPS-stimulated cells used as a positive control.

Next, JAWSII activation mediated by polymer/CpG glycoplexes alone was compared with that mediated by mixed (polymer/CpG + polymer/pOVA) glycoplexes. We used the plasmid encoding ovalbumin as a model antigen to be delivered in association with the CpG adjuvant. While a 4 h pulse of polymer/CpG glycoplexes followed by 20 h chase did not significantly enhance DC activation (Figure S9), significantly higher levels of cytokine release, indicative of a stronger DCs response, were observed when JAWSII cells were coincubated with mixed formulations of (polymer/CpG + polymer/pOVA) glycoplexes (Figure 8A,B).

**Figure 8:**
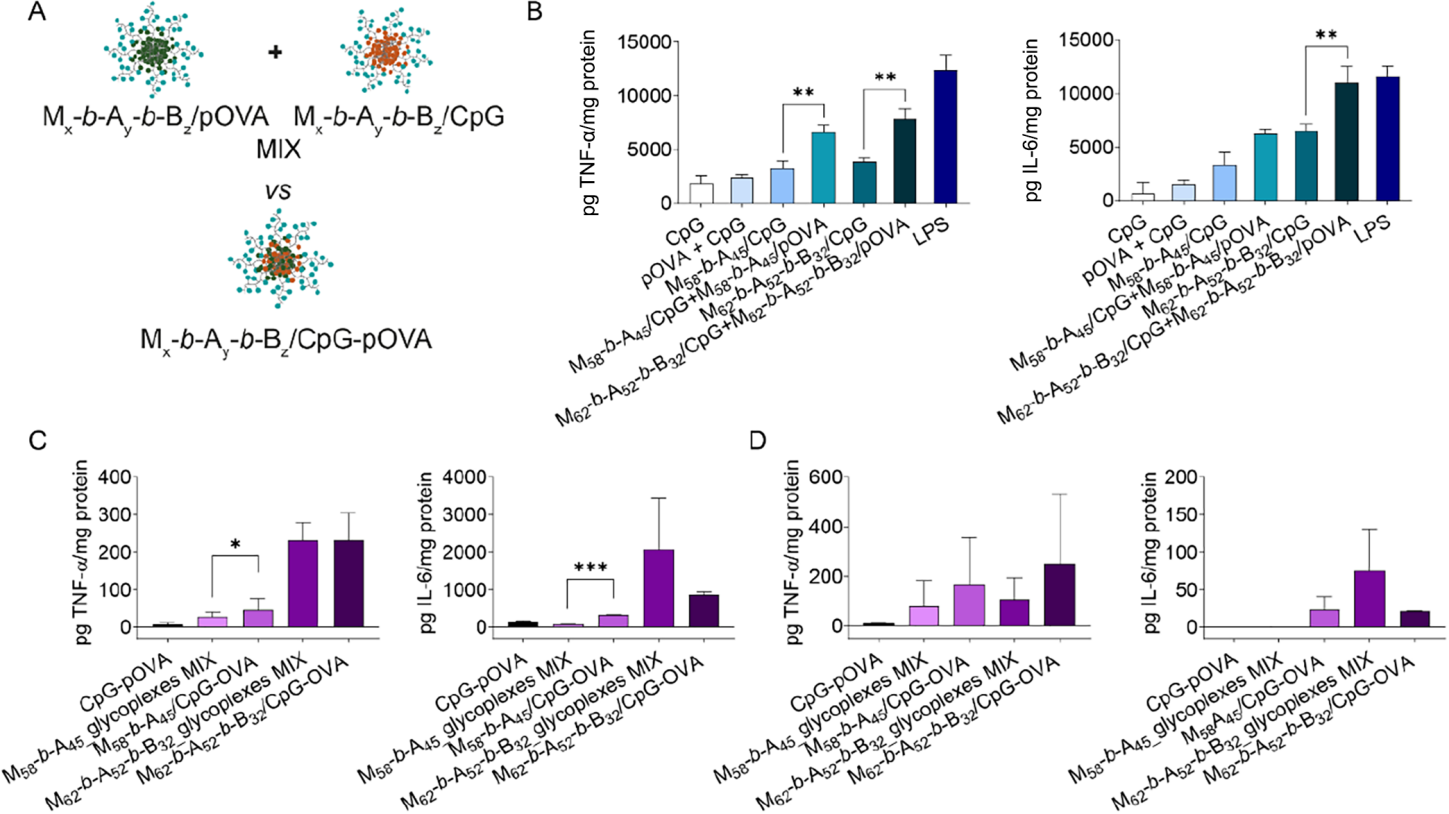
(A) Visual representation of (M_*x*_-*b*-A_*y*_-*b*-B_*z*_/CpG + M_*x*_-*b*-A_*y*_-*b*-B_*z*_/pOVA) and M_*x*_-*b*-A_*y*_-*b*-B_*z*_/CpG-pOVA glycoplexes. In panels (C) and (D), formulations of (M_*x*_-*b*-A_*y*_-*b*-B_*z*_/CpG + M_*x*_-*b*-A_*y*_-*b*-B_*z*_/pOVA) are abbreviated as M_*x*_-*b*-A_*y*_-*b*-B_*z*__glycoplexes MIX. (B–D) JAWSII dendritic cell activation by glycoplexes after 24 h transfection as detected by TNF-α and IL-6 cytokine secretion (panels B and C) or quantified in the lysates (panel D). (B) Coadministration of polymers/CpG with polymers/pOVA glycoplexes was compared with CpG-pOVA alone, or polymer/CpG glycoplex. Untreated cells were used as controls. (C, D) Glycoplexes formulated with CpG and pOVA separately or coformulated within the same glycoplex were compared. Untreated cells were used as controls and their values subtracted. Data are reported as averages ± sd of two independent experiments performed in triplicate (*n* = 2, *p < 0.05, **p < 0.01, ***p < 0.001).

Finally, we compared the activation of JAWSII cells induced by CpG combined with pOVA, either formulated separately into glycoplexes and then mixed (M_*x*_-*b*-A_*y*_-*b*-B_*z*_/CpG + M_*x*_-*b*-A_*y*_-*b*-B_*z*_/pOVA – M_*x*_-*b*-A_*y*_-*b*-B_*z*_ glycoplexes MIX), or coformulated within the same glycoplex (M_*x*_-*b*-A_*y*_-*b*-B_*z*_/CpG-pOVA) (Figure 8A). Again, the new formulations were first characterized by complexation, size, and morphology. Complete complexation of both nucleic acids was confirmed by gel electrophoresis performed on M_*x*_-*b*-A_*y*_-*b*-B_*z*_/CpG-pOVA glycoplexes. Specifically, 12% polyacrylamide and 1% agarose gels were used to distinguish between unbound (smear in the gel) and condensed (nucleic acid retained in the well) forms of CpG and pOVA, respectively (Figure S10A,B). Surprisingly, complete cocomplexation of CpG and pOVA was already observed at an N/P ratio of 2, where the P represents the total number of phosphate groups from both nucleic acids (Figure S10A). TEM and DLS analyses showed that the morphology and PDI of the coformulated glycoplexes were similar to those of polymer/CpG glycoplexes (Figure S10C,D).

To compare DC activation induced by coformulated nucleic acids (M_62_-*b*-A_52_-*b*-B_32_/CpG-pOVA) to that of mixed (M_62_-*b*-A_52_-*b*-B_32_/CpG + M_62_-*b*-A_52_-*b*-B_32_/pOVA) glycoplexes of individual TNAs, we quantified TNF-α and IL-6 production after 24 h of stimulation with both formulations (Figure 8C,D). Data revealed that M_58_-*b*-A_45_/CpG-pOVA induced a stronger JAWSII cell activation compared to (M_58_-*b*-A_45_/CpG + M_58_-*b*-A_45_/pOVA), as indicated by higher cytokine titers in both supernatants and cell lysates.

In contrast, M_62_-*b*-A_52_-*b*-B_32_/CpG-pOVA glycoplexes and (M_62_-*b*-A_52_-*b*-B_32_/CpG + M_62_-*b*-A_52_-*b*-B_32_/pOVA) glycoplexes showed no substantial differences in terms of TNF-α production. However, the mixed formulation of the two separate nucleic acids (M_62_-*b*-A_52_-*b*-B_32_/CpG + M_62_-*b*-A_52_-*b*-B_32_/pOVA) was more effective at inducing IL-6 production compared to M_62_-*b*-A_52_-*b*-B_32_/CpG-pOVA. This finding was unexpected, as studies have previously shown that codelivery of nucleic acids leads to improved efficiency compared to separate coadministration, possibly due to more synchronous cellular uptake.^53,54^ One could speculate that the different behavior observed in our work may be due to a lower ability of M_62_-*b*-A_52_-*b*-B_32_ to complex both nucleic acids within a single particle, which in turn may be indicative of relatively unstable glycoplexes. This would be supported by the results from the gel retardation assay, showing the presence of uncomplexed pOVA at N/P = 1 for M_62_-*b*-A_52_-*b*-B_32_/CpG-pOVA glycoplexes.

Based on these results, glycoplexes derived from M_*x*_-*b*-A_*y*_-*b*-B_*z*_ triblock copolymer emerged as better candidates than those from M_*x*_-*b*-A_*y*_ diblock copolymers for DC activation, potentially due to their faster endosomal escape and nuclear localization, as illustrated in Figure 6B, which is particularly important for pOVA transfection. Overall, all glycoplexes were more effective in stimulating JAWSII cell activation in comparison with CpG-pOVA alone without a carrier.

## 3 Conclusions

This work investigates the codelivery of CpG oligodeoxynucleotides (CpG ODN) and plasmid encoding a model antigen (pOVA) to activate dendritic cells (DCs) in the context of cancer vaccination, using nucleic acid-polycation mannosylated glycoplexes designed to target the endocytic receptor CD206 on DCs. To this aim, we used a novel class of mannosylated copolymers comprising a mannosylated block for targeting dendritic cells (DCs) via endocytic mannose receptor (CD206), an agmatinyl block for nucleic acid condensation in diblock copolymers, and, for a subfamily of these materials, a hydrophobic poly­(butyl acrylate) block to promote the endosomal escape.

From a materials science perspective, this study revealed that, while in general glycosylated ligands with higher valency—i.e., a larger number of sugar repeating units for glycopolymers—tend to have higher avidity for lectin receptors,^55–57^ different binding modalities can be observed when these glycomaterials form part of glycoplexes. Accordingly, data presented in this work show the highest CD206^+^ cell-specific association for ssDNA glycoplexes formed from short M_15_-*b*-A_12_ block copolymers, while the progressive increase in the length of both M_*x*_ and A_*y*_ blocks, as observed in M_29_-*b*-A_25_/ssDNA and M_58_-*b*-A_45_/ssDNA glycoplexes, decreases cell selectivity in cells with high CD206 expression (CHO-CD206^+^). Our molecular dynamics (MD) experiments suggest that chain–chain entanglement of longer mannosylated M_*x*_ blocks can occur at the surface of these ssDNA glycoplexes, which could reduce the ability of these ligands to interact with CD206^+^ cells.

Hydrophobic groups are often included within nucleic acid polyplexes to favor endosomal escape following cell uptake and enhance their biological efficacy.^21,22^ In this work, this was done by introducing a hydrophobic block in the complexing copolymers, to give M_*x*_-*b*-A_*y*_-B_*z*_ triblock copolymers. Results showed a dual effect of such a modification. On one hand, this induced a lower selectivity for the association of glycoplexes with CD206^+^ cells. This reduction in selectivity suggests that for M_*x*_-*b*-A_*y*_-*b*-B_*z*_ copolymers, hydrophobic interactions, rather than receptor-mediated binding mechanisms, may be the main drivers for glycoplex–plasma membrane association. On the other hand, the presence of these hydrophobic poly­(*n*-butyl acrylate) B_z_ blocks improved the intracellular trafficking of the nucleic acid complexes—i.e., these enhanced both the efficiency of endosomal escape and promoted nuclear localization of their nucleic acid cargo. Thus, although the triblock copolymers were less selective at targeting CD206-expressing cells, their higher ability to promote endosomal escape and nuclear delivery may make them a more effective delivery system in settings where precise cellular targeting is less critical. This highlights a valuable trade-off between targeting specificity and delivery efficiency that could be exploited when needed for specific therapeutic applications. Taken together, these results provide a structure–function relationship for this new class of glycosylated complexing copolymers and suggest that, for these materials and potentially for a range of structurally analogous materials, optimization of nucleic acid complexation and delivery requires a fine balance of the copolymer structural parameters, i.e., size, hydrophobic/hydrophilic balance, overall charge, and sugar valency.

From a therapeutic point of view, our findings demonstrate that mannosylated M_*x*_-*b*-A_*y*_ diblock and M_*x*_-*b*-A_*y*_-B_*z*_ triblock copolymers effectively condense ssDNA and enable the delivery of immunostimulatory CpG to dendritic cells, leading to enhanced DC activation compared to free CpG. Moreover, codelivery of CpG and a plasmid encoding the model antigen ovalbumin (pOVA), whether encapsulated within the same polyplex or administered as separate formulations, elicited distinct DC activation profiles. This underscores the potential of this block copolymer for tailored nucleic acid delivery in cancer vaccination and immunotherapy.

## 4 Experimental Section

### 4.1 Materials

Acrylamide/bis acrylamide 30:1 solution, glycerol, TEMED, TMB, Triton X-100, Tween 20, xylene cyanol FF, thiazolyl blue tetrazolium bromide (MTT), and Triton X-100 were obtained from Sigma-Aldrich (St. Louis, MO) and Fisher Scientific (Hampton, NH). Organic solvents, salts, and buffers were purchased from Sigma-Aldrich (St. Louis, MO) and Fisher Scientific (Hampton, NH). 19-Base single-stranded DNA (ssDNA) (5′-GAGATGTAAGGCCAGGCCG-3′), cyanin-3 labeled ssDNA (cy3-ssDNA), and cyanin-5 labeled ssDNA (cy5-ssDNA) were supplied by BIOMERS.NET GmbH (Ulm, Germany). CpG ODN 1585 was supplied by InvivoGen (San Diego, CA). Ovalbumin-encoding plasmid (pOVA) was purchased from GeneScript (Leiden, Netherlands) and amplified by *Escherichia coli* bacterial cell transformation using One Shot TOP10 chemically competent *E. coli* (Invitrogen, Carlsbad) and purified with a NucleoBond Xtra Maxi kit (Macherey-Nagel GmbH, Düren, Germany). GelRed nucleic acid staining solution 10,000× in water was purchased from Biotium (Fremont, CA). Purified anti-mouse CD16/32 antibody (clone 93), Alexa Fluor 647 anti-mouse CD206 Antibody, Alexa Fluor 647 Rat IgG2a, k Isotype Ctrl, and Alexa Fluor 647-conjugate goat anti-Rat IgG were purchased from Biolegend (San Diego, CA). Rabbit anti-mouse anti-EEA-1 primary antibody and Alexa Fluor 488 goat anti-rabbit IgG (H + L) secondary antibody were purchased from Cell Signaling Technology (Danvers, MA). Rat anti-mouse LAMP-1 primary antibody, cyanine-3 goat anti-rat IgG secondary antibody, PE rat anti-mouse I-A/I-E MHC II, Alexa Fluor 647 rat anti-mouse CD86, FITC hamster anti-mouse CD80, Alexa Fluor 647 rat anti-mouse CD40, Alexa Fluor 647 rat anti-mouse CD206 and their isotype controls PE rat IgG2b, κ, Alexa Fluor 647 rat IgG2a, κ and FITC hamster IgG were supplied by BioLegend (San Diego, CA). Mouse TNF-α and IL-6 ELISA detection kits were purchased from R&D SYSTEM (Minneapolis, MN). Lipopolysaccharide (LPS), fetal bovine serum (FBS), Dulbecco’s modified Eagle medium (DMEM), Ham’s F12 medium (F12), Opti-MEM reduced serum medium, Roswell Park Memorial Institute (RPMI 1640) medium, non-essential amino acids 100× solution, β-mercaptoethanol, l-glutamine, penicillin/streptomycin solution, Geneticin (G418) disulfate salt, trypsin solution, phosphate-buffered saline (PBS) with and without calcium and magnesium, paraformaldehyde (PFA), 4′,6-diamidino-2-phenylindole (DAPI), and all the other reagents for cell culture were purchased from Sigma-Aldrich (St. Louis, MO), Fisher Scientific (Hampton, NH) or Gibco Thermo Fisher Scientific (Waltham, MA). CHO (wild type Chinese Hamster ovarian cell line), CHO-CD206^+^ (mannose receptor expressing CHO cell line), and DC2.4 cells were kindly donated by Prof. Luisa Martinez-Pomares (Faculty of Medicine & Health Sciences, University of Nottingham). JAWSII cell line was purchased from American Type Culture Collection (CRL-1194; ATCC, Manassas, VA).

### 4.2 Glycoplexes Formulation and Electrophoretic Mobility Shift Assays (EMSAs)

M_*x*_-*b*-A_*y*_ diblock and M_*x*_-*b*-A_*y*_-*b*-B_*z*_ triblock copolymers were generated as already described by our group, and their ^1^H NMR and gel permeation chromatography (GPC) characterizations were first reported in Bellato et al. (Figures S11–S16).^11^ Copolymer solutions in the 0.05–5 mg mL^–1^ concentration range were prepared in Milli-Q water, and glycoplexes were assembled by using model ssDNA, CpG ODN 1585, pOVA, or a combination of CpG + pOVA according to the procedures described below.

#### 4.2.1 Copolymer/ssDNA Glycoplexes Formulation

Copolymer aliquots in Milli-Q water at the appropriate concentration were added to a fixed concentration of 19-base ssDNA (0.52 μL, 300 ng, 0.52 pmol, 100 μM solution) to achieve nitrogen/phosphate (N/P) molar ratios in the 1–20 range. The solution was gently mixed with pipetting, incubated for 1 h at room temperature, and diluted to a final volume of 10 μL with PBS.

#### 4.2.2 Copolymer/CpG Glycoplexes Formulation

Copolymer aliquots in Milli-Q water at the appropriate concentration were added to a fixed concentration of CpG ODN 1585 (3 μL, 300 ng, 47 pmol, 15 μM solution) to achieve nitrogen to phosphate (N/P) molar ratios in the 1–20 range. The solution was gently mixed by pipetting, incubated for 1 h at room temperature, and diluted to the final volume of 10 μL with Milli-Q water.

#### 4.2.3 Copolymer/CpG-pOVA Formulation

Copolymer aliquots in Milli-Q water at the appropriate concentration were added to a fixed concentration of CpG ODN 1585 (3 μL, 300 ng, 47 pmol, 15 μM solution) and pOVA (1 μL, 100 ng, 0.0246 pmol) to achieve nitrogen to phosphate (N/P) molar ratios in the 1–10 and 0.1–10 ranges for M_58_-*b*-A_45_ and M_62_-*b*-A_52_-*b*-B_32_, respectively, gently mixed by pipetting, incubated for 1 h at room temperature, and diluted to the final volume of 10 μL with Milli-Q water.

Copolymer/DNA complexes were then analyzed by either:

- 12% v/v polyacrylamide gel run at 100 V for 50 min using TBE (0.1 M Tris base, 0.1 M boric acid, 0.002 M ethylenediaminetetraacetic acid (EDTA)) as running buffer.
- 1% w/v agarose gel electrophoresis at 100 V for 45 min using Tris-ammonium-ethylenediaminetetraacetic acid (TAE, 40 mM Tris base, 40 mM acetic acid, 1 mM EDTA) as running buffer.

The bands corresponding to ssDNA, CpG, or pOVA were visualized under ultraviolet light after staining by immersing the gel for 30 min in 50 mL of Milli-Q water containing 10 μL of 10,000× GelRed nucleic acid gel staining. Images of gels were acquired with an Azure 400 bioanalytical imaging system (Azure Biosystems, CA).

### 4.3 Heparin Displacement Assay

Copolymer/ssDNA polyplexes were prepared at the N/P ratios selected in Section 4.2. Glycoplexes (300 ng of ssDNA) were incubated for 15 min with increasing concentration of heparin (0.15–10 IU mL^–1^) and loaded into a 12% v/v polyacrylamide gel. The conditions used for gel preparation, running, and visualization were as reported in the previous section.

### 4.4 Copolymer/DNA Complexes Characterizations

The mean particle diameter and polydispersity index (PDI) of the glycoplexes prepared at the N/P ratio that showed full ssDNA complexation by electrophoretic mobility shift assays were determined by dynamic light scattering (DLS) using Malvern Instrument Ltd. (Malvern Instrument Ltd., U.K.) at a constant scattering angle of 173° and at 25 °C and a DNA concentration of 24 μg mL^–1^. The zeta potential (ζ) of copolymer/ssDNA glycoplexes was investigated by laser Doppler electrophoresis using Zetasizer Nano ZS (Malvern, U.K.) at a fixed temperature of 25 °C and a copolymer concentration of 0.1 mg mL^–1^ in 5 mM HEPES, pH 7.4. The average values of size and ζ potential are reported as the average of three measurements ± sd. Polyplexes morphology was evaluated in negative staining mode by transmission electron microscopy, using a Tecnai G2 microscope (FEI, OR). Samples were prepared in PBS or Milli-Q water at a final polymer concentration of 0.25 mg mL^–1^ and at the same N/P ratios used for the DLS analysis and were deposited on a small holey carbon-coated support grid (400 mesh). The excess volume was removed with filter paper and then stained using 1% w/v uranyl acetate aqueous solution as a contrast agent. The average diameter of glycoplexes and the percentage of spherical, rod- or toroid-shaped complexes were evaluated by ImageJ software v.1.51 by measuring 50 individual polyplexes.

### 4.5 Glycoplexes Stability in Media Mimicking Physiological Conditions

Polyplexes samples were prepared by mixing 4.16 μL of 100 μM ssDNA (2.4 μg) with M_15_-*b*-A_12_ (77 μg, N/P 20), M_29_-*b*-A_25_ (36.4 μg, N/P 10), M_58_-*b*-A_45_ (18.3 μg, N/P 5), M_29_-*b*-A_29_-*b*-B_9_ (10.8 μg, N/P 3), and M_62_-*b*-A_52_-*b*-B_32_ (13.2 μg, N/P 3) polymers solution in Milli-Q water. After mixing, the samples were equilibrated at room temperature for 1 h and then (a) dilute to 100 μL with PBS and then analyzed at regular intervals over a 2-week period, storing the solutions at 4 °C or (b) diluted to 100 μL with PBS added of 10% fetal bovine serum and incubated at 37 °C, analyzing the samples at scheduled times over 48 h.

### 4.6 Molecular Dynamic Simulations

Molecular dynamics (MD) simulations were carried out using the all-atom optimized potentials for liquid simulations (OPLS-AA) force field (both for ssDNA and copolymers).^58,59^ The force field parameters for the units of the copolymers were obtained using the LigparGen web server.^60^ For water, the TIP3P^61^ model and for Cl^–^ the model by Chandrasekhar et al.^62^ were employed. The GROMACS simulation package^63^ was used.

We performed simulations of (i) single copolymers (1 M_58_-*b*-A_45_ or 1 M_62_-*b*-A_52_-*b*-B_32_ in 230,000 water molecules) and (ii) 1 ssDNA filament (the same sequence used in experiments, that is, 5′-GAGATGTAAGGCCAGGCCG-3′) with various numbers of copolymers and water molecules (30 M_15_-*b*-A_12_/120,000 H_2_O, 8 M_29_-*b*-A_25_/230,000 H_2_O, 2 M_29_-*b*-A_29_-*b*-B_9_/230,000 H_2_O). All systems were neutralized with Cl^–^ ions. Periodic boundary conditions were employed. The leapfrog integrator was used, with a time step of 2 fs, and bonds involving hydrogens were constrained using the LINCS algorithm.^64^ Long-range electrostatic interactions were treated with the particle mesh Ewald (PME)^65^ method, and the Lennard-Jones potential was truncated at 1.0 nm.

All simulations started from the random positions of the molecules in the simulation box. After energy minimization, a 1 ns trajectory in the NVT (*T* = 300 K) ensemble was run, using the Berendsen thermostat.^66^ Then, a simulation in the NPT ensemble (*T* = 300 K, *p* = 1 bar) was carried out, using the V-rescale thermostat^67^ and the Parrinello–Rahman^68^ barostat (500 ns). During the trajectories of mixed systems, clusters containing DNA and a certain number of copolymers were formed (Figure 2 in the main text).

### 4.7 Ex Vivo Hemolysis Assay of Free Copolymers and Assembled Glycoplexes

Isolation of Red Blood Cells (RBCs) was performed as reported by Evans et al.^69^

Briefly, 5 mL of rat blood was centrifuged at 500*g* for 5 min at 4 °C. The supernatant was removed, and the pellet was washed three times with saline solution (0.9% w/v NaCl) and once with PBS. After the last washing step, the cell pellet was resuspended in PBS, pH 7.4, or MES (10 mM MES, 149 mM NaCl), pH 6.5, at 2% v/v concentration. 180 μL aliquots of RBCs suspension were mixed with (a) 20 μL of 0.05–10 mg mL^–1^ polymer solutions, or with (b) 0.05–10 mg mL^–1^ polymer concentration in glycoplexes, to achieve 5–1000 μg mL^–1^ final polymer concentrations. Samples were incubated at 37 °C for 1 h and then centrifuged at 500*g* for 5 min. 100 μL of the supernatant was transferred to a 96-well plate, and the absorbance of the released hemoglobin was detected at 490 nm with an ELISA plate reader (Bio-TEKTM Instruments, Inc. EL 3II5X (Winooski)). The percentage of hemolysis was calculated according to eq 1:

1 $$\%\;\mathrm{hemolysis}=\frac{{\mathrm{OD}}_{\mathrm{sample}}-{\mathrm{OD}}_{\mathrm{negative}\;\mathrm{CTRL}}}{{\mathrm{OD}}_{\mathrm{positive}\;\mathrm{CTRL}}-{\mathrm{OD}}_{\mathrm{negative}\;\mathrm{CTRL}}}\times 100$$

where negative CTRL are PBS pH 7.4 or MES pH 6.5, and positive control is 1% Triton X-100 considered as 100% of hemolysis.

### 4.8 In Vitro Studies

Cell lines were grown at 37 °C in a humidified atmosphere with 5% CO_2_. CHO and CHO-CD206^+^ cells were cultivated in Ham’s F12/Dulbecco Modified Eagle Medium (DMEM) (50:50) supplemented with 10% FBS, 2 mM l-glutamine, 100 U mL^–1^ penicillin, and 100 μg mL^–1^ streptomycin (DMEM/F12 complete medium). CHO-CD206^+^ cells were added to 0.6 mg mL^–1^ Geneticin to maintain clone selection. DC2.4 cell line was cultivated using Roswell Park Memorial Institute (RPMI 1640) medium supplemented with 10% FBS, 2 mM l-glutamine, 100 U mL^–1^ penicillin, 100 μg mL^–1^ streptomycin, 1% non-essential amino acid, and 50 μM β-mercaptoethanol (RPMI complete medium). JAWSII cells were grown in α-minimal essential medium (MEM) supplemented with 20% FBS, 4 mM l-glutamine, 100 U mL to 1 penicillin, 100 μg mL^–1^ streptomycin, and 5 ng mL^–1^ granulocyte-macrophage colony-stimulating factor (GM-CSF, α-MEM complete medium).

#### 4.8.1 Glycoplexes Biocompatibility Studies

To evaluate the glycoplexes biocompatibility, CHO and CHO-CD206^+^ cells were seeded in a 96-well plate (1 × 10^4^ cells per well) in DMEM/F12 complete medium, and after overnight culture, the medium was replaced with 100 μL of copolymer/ssDNA suspensions prepared in DMEM/F12 as described in Section 4.2. Glycoplexes were prepared in the 125–1000 nM ssDNA concentration range using the N/P ratios of 20, 10, and 5 for M_15_-*b*-A_12_/ssDNA, M_29_-*b*-A_25_/ssDNA, M_58_-*b*-A_45_/ssDNA, respectively, and of 3 for both M_29_-*b*-A_29_-*b*-B_9_/ssDNA and M_62_-*b*-A_52_-*b*-B_32_/ssDNA. After 24 h incubation, cells were washed with 2× 100 μL PBS and the cell survival was assessed through 3-(4,5-dimethylthiazol-2-yl)-2,5-diphenyl tetrazolinium bromide (MTT) assay.^70^ 180 μL of serum-free medium was added to each well followed by 20 μL of MTT solution (5 mg mL^–1^ in PBS). After 3 h of incubation at 37 °C, the medium was removed and replaced with 200 μL of dimethyl sulfoxide (DMSO). Plates were left 15 min under gentle shaking to allow formazan crystal dissolution and then analyzed via spectrophotometric measurement at 570 nm. The cell viability was calculated according to eq 2 using cell incubated with medium only as positive control (100% viability).

2 $$\mathrm{cell}\;\mathrm{viability}\;\left(\%\right)=\frac{{\mathrm{OD}}_{\mathrm{sample}}-{\mathrm{OD}}_{\mathrm{CTRL}}}{{\mathrm{OD}}_{\mathrm{CTRL}}}\times 100$$

#### 4.8.2 Copolymer/ssDNA Glycoplexes Association with Cells Expressing CD206

Copolymer/ssDNA association with cells was evaluated in Chinese hamster ovary cells (CHO and CHO-CD206^+^) and in immortalized dendritic cells (DC2.4). Glycoplexes were prepared by using cyanine-3 (cy3)-labeled ssDNA (cy3-ssDNA).

CHO and CHO-CD206^+^ cells were seeded at a density of 2 × 10^5^ cells per well in complete DMEM/F12, while DC2.4 cells were seeded at a density of 5 × 10^4^ cells per well in complete RPMI 1640 medium in a 24-well plate. After overnight culture, cells were washed twice with 500 μL of PBS and added of copolymer/cy3-ssDNA glycoplexes suspension (400 μL) prepared in serum-free DMEM/F12 (for CHO and CHO-CD206^+^) or RPMI (for DC2.4) media as described in Section 4.2, at a 125 nM cy3-ssDNA fixed concentration. After either 30 min or 1 h of incubation, glycoplexes containing medium were removed, and cells were washed twice with 500 μL PBS. Cells were harvested by 2 min incubation with 100 μL of a 0.06% w/v trypsin solution in PBS followed by the addition of 200 μL of PBS with Ca^2+^ and Mg^2+^. Cells were transferred into flow cytometry tubes, fixed with 100 μL of a 4% v/v paraformaldehyde (PFA) solution in PBS, and analyzed with FC500 Beckmann Coulter flow cytometry with CXP acquisition (Becton, Dickinson and Company, Buccinasco, Milan). The mean fluorescence intensity was detected on FL2 (argon laser: 488 nm excitation, 575–540 nm filter detection). At least 1 × 10^4^ events in the gate were recorded per sample. Data were analyzed with Flowing software v.2.5.1 (Turku Centre for Biotechnology, Finland).

#### 4.8.3 CD206 Surface Expression

Prior to carrying out the in vitro experiments, the presence of CD206 at the cell membrane of CHO/CHO-CD206^+^, DC2.4, and JAWSII cell lines was assessed by immunostaining with Alexa Fluor 647 anti-mouse CD206 Antibody.

1 × 10^7^ cells were resuspended in 1 mL of blocking buffer (5% inactivated goat serum, 0.5% bovine serum albumin (BSA), 2 mM NaN_3_, 5 mM EDTA in PBS) containing 1:50 dilution of Anti-CD16/CD32 antibody (Biolegend), aliquoted into 100 μL samples, and incubated on ice for 30 min.

Cells were then centrifuged at 1000 rpm using a benchtop centrifuge, for 5 min at 4 °C, resuspended in 100 μL of blocking buffer containing 1 μL of Alexa Fluor 647 anti-mouse CD206 antibody or isotype control (Alexa Fluor 647 Rat IgG2a,κ Isotype Ctrl) and incubated for 1 h at 4 °C. Cells were washed three times with 0.5 mL of FACS buffer (0.5% BSA, 2 mM sodium azide, 5 mM EDTA in PBS) and resuspended in 400 μL of FACS buffer containing 1% paraformaldehyde. Cells were analyzed on a BD FACSAria III Cell Sorter with CXP acquisition (Becton, Dickinson and Company, Buccinasco, Milan). 2 × 10^4^ events were acquired for each sample, and the fluorescence was recorded on APC channel (λ_ex_ 650 nm, λ_em_ 665 nm). Untreated cells were also analyzed to estimate cell autofluorescence. Data were analyzed with Flowing software v.2.5.1 (Turku Centre for Biotechnology, Finland).

#### 4.8.4 CpG and CpG-pOVA Effect on the Surface Expression of Cluster of Differentiation by JAWSII Cells

To evaluate CpG ODN 1585 activity as adjuvant, JAWSII cells were seeded in a 24-well plate at a density of 2 × 10^5^ cells per well in complete αMEM medium supplemented with 5 ng mL^–1^ GM-CSF. After overnight incubation, cells were washed twice with 500 μL PBS and added 400 μL of M_58_-*b*-A_45_/CpG and M_62_-*b*-A_52_-*b*-B_32_/CpG complex suspension prepared at the N/P ratio of 3 in Opti-MEM + 1% v/v FBS, as described in Section 4.2, and at a 2.5 μg mL^–1^ ODN 1585 concentration. Medium only and CpG ODN 1585 alone were used as controls. After 24 h incubation, the medium was discarded, and cells were rinsed with 500 μL of PBS followed by incubation with 25 mM EDTA solution in PBS for 8 min at 37 °C. Cells were collected by centrifugation at 1500 rpm for 5 min at 4 °C, resuspended in 50 μL of blocking buffer (2 mM NaN_3_, 5 mM EDTA, 5% v/v FBS, 0.5% w/v BSA in PBS), and incubated at 4 °C for 30 min. Then, cells were added with 50 μL of blocking buffer containing different antibodies: (a) PE-anti-mouse IA/I-E MHC II rat Ab (2 μg mL^–1^); (b) AlexaFluor647-anti-mouse CD86 rat Ab (0.5 μg mL^–1^); (c) FITC-anti mouse CD80 rat Ab (6.5 μg mL^–1^); (d) AlexaFluor647-anti mouse CD40 Ab (2 μg mL^–1^). Alternatively, cells were added of corresponding antibody isotype solutions: (a) PE-IgG2b,κ rat Ab (2 μg mL^–1^); (b, d) AlexaFluor647-IgG2a,κ rat Ab (0.5 or 2 μg mL^–1^); (c) FITC-IgG Armenian Hamster Ab (6.5 μg mL^–1^). After 1 h of incubation at 4 °C, 900 μL of PBS was added and cells were centrifuged at 1500 rpm and 4 °C for 5 min and rinsed with 2× 500 μL FACS buffer (2 mM NaN_3_, 5 mM EDTA, 0.5% w/v BSA in PBS). After the last washing step, cells were resuspended in 300 μL FACS buffer, fixed with 100 μL 4% PFA solution in PBS, and transferred into FACS tube for analysis. Samples were analyzed on a BD FACSAria III Cell Sorter with CXP acquisition (Becton, Dickinson and Company, Buccinasco, Milan). The mean fluorescence intensity was detected on PE (excitation by 488 nm laser, 575–540 nm detection range) and APC (excitation: λ = 633 nm laser, 660–620 nm detection range) channels. At least 1 × 10^4^ events in the gate were recorded per sample. Untreated cells not stained with Ab and untreated cells stained with Ab were used as controls. Data were analyzed with Flowing software v.2.5.1 (Turku Centre for Biotechnology, Finland).

#### 4.8.5 Glycoplexes Cellular Trafficking

DC2.4 cells were seeded at a density of 4 × 10^4^ cells per well in 4-well BD Falcon Culture Slide and were grown overnight in RPMI 1640 + 10% v/v FBS. Then, cells were rinsed and added with 500 μL of M_58_-*b*-A_45_/cy5-ssDNA or M_62_-*b*-A_52_-*b*-B_32_/cy5-ssDNA glycoplexes (125 nM cy5-ssDNA) at an N/P ratio of 5 and 3, respectively. After 15 and 30 min of incubation at 37 °C in a humidified atmosphere with 5% CO_2_, the cells were rinsed with 3× 500 μL PBS and immediately fixed by incubation with 4% PFA in PBS for 15 min at 4 °C or further incubated for 2 h with fresh medium and then fixed as described. Then, cells were washed thrice with 500 μL PBS and were permeabilized by incubation with 200 μL of 5% v/v FBS, 0.25% v/v Triton X-100 in PBS for 45 min in the dark at room temperature. Afterward, the solution was discarded, and samples were washed with 3× 500 μL PBS followed by the addition of 200 μL of primary antibodies (a) anti-mouse anti-EEA-1 (1:100 dilution in 5% v/v FBS in PBS) or (b) anti-mouse anti-LAMP-1 (1:100 dilution in 5% v/v FBS in PBS). After 1 h incubation at room temperature in the dark, the cells were rinsed with 3× 500 μL PBS, and then the nuclei were stained by incubation with 200 μL of a 2 μg mL^–1^ DAPI solution in PBS, followed by incubation with secondary antibodies (a) cy3-goat anti-rat IgG (1:200 dilution), or (b) Alexa Fluor 488 goat anti-rabbit IgG (H + L) (1:200 dilution). Finally, samples were washed with 3× 500 μL of PBS and chambers were mounted on glass slides using Mowiol aqueous mounting media. Cells were imaged with a Zeiss confocal laser scanning microscope (LSM 800, Zeiss, Jena, Germany) using an immersion lens with 63× magnification, with wavelengths at 410–540 nm (blue, DAPI) for nuclei detection, 488–530 nm for Alexa Fluor 488 (green), and 645–700 nm for cy5-ssDNA detection (red). Untreated cells or cells treated with cy5-ssDNA alone were used as controls.

The images were then processed with ZEN 3.9 (blue edition) from Zeiss Software.

#### 4.8.6 Evaluation of Glycoplexes Endosomal Escape Properties by Förster Resonance Energy Transfer

CHO-CD206^+^ cells were seeded at a density of 2.5 × 10^4^ cells per well on a 24-well plate containing glass dishes and were grown overnight. Cells were then incubated at 37 °C with 400 μL of M_58_-*b*-A_45_/ssDNA glycoplexes at an N/P ratio of 5 and prepared as described in Section 4.2 in serum-free DMEM/F12 using a mix of cy3- and cy5-ssDNA, at 125 nM concentration. After 2 h incubation, cells were washed with 2× 400 μL of PBS and then incubated with DMEM/F12 + 10% FBS for 2, 6, or 24 h. Then, cells were washed with 3× 400 μL of PBS and were fixed by incubation with 300 μL of a 4% paraformaldehyde (PFA) solution in PBS for 15 min at room temperature. Afterward, the PFA solution was discarded, and cells were rinsed with 3× 400 μL PBS, followed by nuclei staining with 300 μL of a 4.5 μg mL^–1^ DAPI solution in PBS for 10 min. Finally, glass dishes were gently rinsed with 3× 300 μL of PBS and once with 300 μL of Milli-Q water before being mounted on microscope slides using Mowiol aqueous mounting media.

Cells were imaged with a Zeiss confocal laser scanning microscope (LSM 800, Zeiss, Jena, Germany) using an immersion lens with 63× magnification, with wavelengths at 410–540 nm (blue, DAPI) for nuclei detection, 540–612 nm for cy3-ssDNA detection (green, donor), and 645–700 nm for cy5-ssDNA detection (red, acceptor). These settings were applied for image acquisition before and after a high-power laser was applied for cy5 probe bleaching. The images were then processed with ZEN 3.9 (blue edition) from Zeiss Software. The increase in cy3 emission after cy5 bleaching due to a decrease of Förster resonance energy transfer (FRET) was calculated as energy transfer efficiency (Eff_FRET_) for the endosomal escape evaluation according to the following equation:

3 $${\mathrm{Eff}}_{\mathrm{FRET}}=\frac{D_{\mathrm{AB}}-D_{\mathrm{BB}}}{D_{\mathrm{AB}}}$$

with *D*_BB_ and *D*_AB_ referring to the donor fluorescence intensities before and after acceptor photobleaching, respectively.

#### 4.8.7 Stimulation of JAWSII and Cytokines Analysis (Enzyme-Linked Immunosorbent Assay)

##### 4.8.7.1 Cells Stimulation

JAWSII cells were seeded at a density of 2.2 × 10^5^ cells per well in a 24-well plate and were grown for 24 h in complete medium. Then, M_58_-*b*-A_45_/CpG, M_58_-*b*-A_45_/pOVA, M_58_-*b*-A_45_/CpG-pOVA, M_58_-*b*-A_45_/CpG + M_58_-*b*-A_45_/pOVA, M_62_-*b*-A_52_-*b*-B_32_/CpG, M_62_-*b*-A_52_-*b*-B_32_/pOVA, M_62_-*b*-A_52_-*b*-A_32_/CpG-pOVA, and M_62_-*b*-A_52_-*b*-B_32_/CpG + M_62_-*b*-A_52_-*b*-B_32_/pOVA glycoplexes (400 μL, 2.5 and 1.375 μg mL^–1^ ODN 1585 and pOVA final concentrations, respectively) prepared in Opti-MEM + 1% v/v FBS as described in Section 4.2 were added to the cells and incubated for 4 or 24 h at 37 °C, 5% CO_2_. In the case of 4 h incubation, after the incubation, the medium was discarded and replaced with glycoplexes-free medium, followed by further 20 h incubation. Untreated cells, cells treated with 2.5 μg mL^–1^ CpG ODN 1585, 2.5 μg mL^–1^ CpG ODN 1585 + 1.375 μg mL^–1^ pOVA, and cells treated with 10 ng mL^–1^ LPS were used as controls. After 24 h incubation, supernatants were collected, centrifuged at 2000 rpm for 5 min at 4 °C, and aliquoted. Cells were lysed with 300 μL of lysis buffer (10 mM Tris–HCl, 2% Triton X-100, 50 mM NaCl, 2 mM EDTA, pH 8.2) containing protease inhibitors (cOmplete mini EDTA-free). Lysates were collected and centrifuged at 2000 rpm for 5 min at 4 °C, transferred into a new vial, centrifuged again at 12,000 rpm for 30 min at 4 °C, and aliquoted. Samples were stored at −80 °C until analysis. The protein content of each sample was quantified by BCA analysis according to the manufacturer’s instructions.

##### 4.8.7.2 Cytokines Quantification

Each well of a 96-well plate (MaxiSorp, NUNC) was coated with 100 μL of capture antibody diluted to the working concentration (800 ng mL^–1^ for TNF-α and 2 μg mL^–1^ for IL-6) in PBS. The plate was sealed and incubated overnight at room temperature. Then, the capture antibody solution was removed, and wells were rinsed with 3× 300 μL wash buffer (0.05% v/v Tween 20 in PBS). Wells were blocked by adding 300 μL of reagent diluent (1% w/v BSA in PBS, filtered under sterile conditions) and incubating at room temperature for 1 h. Then, 100 μL of cytokine standard solutions in reagent diluent (31.3–2000 ng mL^–1^ concentration range for TNF-α, 15.6–1000 ng mL^–1^ concentration range for IL-6) or 100 μL of samples at the appropriate dilution were added to the wells, and the plate was covered with an adhesive strip and incubated at room temperature. After 2 h, the wash step was repeated, and then 100 μL of detection antibody diluted in reagent diluent to the working concentration (70 ng mL^–1^ for TNF-α and 75 ng mL^–1^ for IL-6) was added to each well. The plate was covered and incubated for a further 2 h. The plate was then washed three times with a wash buffer. Afterward, 100 μL of a 40-fold dilution of streptavidin–horseradish peroxidase (HRP) in reagent diluent was added to each well. The plate was covered and incubated again for 20 min at room temperature in the dark. The wash was repeated, then 100 μL of substrate solution (1:1 mixture of 3,3′,5,5′-tetramethylbenzidine (TMB) and H_2_O_2_) was added to each well. After 20 min of incubation at room temperature in the dark, the enzymatic reaction was stopped by adding 50 μL of stop solution (2 N H_2_SO_4_) to each well.

The optical density of each well was immediately recorded at 450 nm (sample reading) with the correction wavelength set at 570 nm (background measurement) using an ELISA plate reader. Readings at λ = 570 nm were subtracted from those at λ = 450 nm. Sample concentrations were calculated by considering the specific calibration curve.

Cytokine concentration was normalized per protein concentration in samples as detected by the BCA assay.

## Supplementary Material



## Data Availability

The data that support the findings of this study are available upon request from the corresponding author.

## Notes

TEM images of glycoplexes (Figure S1); snapshot from an MD trajectory of M_62_-*b*-A_52_-*b*-B_32_ in water (Figure S2); MTT cell viability on CHO and CHO-CD206^+^ cells (Figure S3); glycoplexes association profile with CHO and CHO-CD206^+^ cells by flow cytometric (FC) analysis (Figure S4); CD206 expression by CHO/CHO-CD206^+^ and DC2.4 cell lines (Figure S5); confocal microscopy images of DC2.4 cell line incubated for 15 or 30 min with M_58_-*b*-A_45_/cy5-ssDNA and M_62_-*b*-A_52_-*b*-B_32_/cy5-ssDNA (Figure S6); cell surface CD206 expression by JAWSII cell line (Figure S7); polymer/CpG characterizations by gel electrophoresis, DLS, and TEM (Figure S8); TNF-α and IL-6 cytokines release by JASWII cells induced by 4 h transfection + 20 h post-transfection with polymers/CpG glycoplexes (Figure S9); characterization M_58_-*b*-A_45_/CpG-pOVA and M_62_-*b*-A_52_-*b*-B_32_/CpG-pOVA glycoplexeplexes by electrophoretic mobility shift assay, DLS, and TEM (Figure S10); ^1^H NMR of purified M_*x*_-*b*-A_*y*_-*b*-B_*z*_ in DMSO-*d*_6_ (Figures S11–S15); and GPC profiles of purified M_*x*_-*b*-A_*y*_-*b*-B_*z*_ (Figure S16) (PDF)
