# Supplementary material for: Mannose Targeting and Hydrophobic Tuning of Polycationic Vectors for Efficient Immunostimulatory CpG Delivery
Source: ACS Appl Nano Mater. 2025 Nov 17;8(47):22637–56. doi: 10.1021/acsanm.5c03883 (PMC12670563; doi:10.1021/acsanm.5c03883)
Supplement: Supplementary file 1 [file an5c03883_si_001.pdf]

Supporting Information

Mannose Targeting and Hydrophobic Tuning of Polycationic Vectors for Efficient Immunostimulatory CpG Delivery

*Federica Bellato,[1†] Greta Bellio,[1] Daniele Asnicar,[2] Rosa Catania,[3,4] Linda Pecchielan,[1] Lara Marcenta,[1] Marco Zanon,[1] Anna Cielo,[1] Marica Zainotto,[5] Marco Pirazzini [5] Alberta Ferrarini,[2] Giuseppe Mantovani,[3] Francesca Mastrotto[1]**

[1]Department of Pharmaceutical and Pharmacological Sciences, University of Padova, Via F. Marzolo 5, 35131 Padova, Italy

[2]Department of Chemical Sciences, University of Padova, Via F. Marzolo 1, 35131 Padova, Italy

[3]School of Pharmacy, University of Nottingham, Nottingham NG7 2RD, U.K.

[4]School of Chemistry, University of Leeds, Leeds LS2 9JT, UK; Astbury Centre for Structural Molecular Biology, University of Leeds, Leeds LS2 9JT, UK

[5]Department of Biomedical Sciences, University of Padova, Via Ugo Bassi 58/B, 35131 Padova, Italy

*Email: francesca.mastrotto@unipd.it

Additional Figures

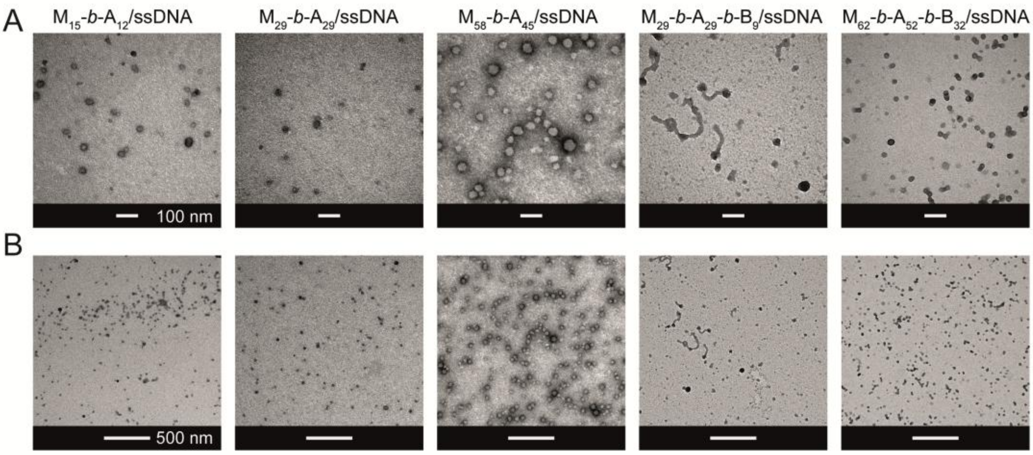

Figure S1. (A,B) Transmission Electron Microscopy (TEM) images of M_{15}-*b*-A_{12}/ssDNA at the N/P of 20, M_{29}-*b*-A_{25}/ssDNA at the N/P of 10, M_{58}-*b*-A_{45}/ssDNA at the N/P of 5, and M_{29}-*b*-A_{29}-*b*-B_9/ssDNA and M_{62}-*b*-A_{52}-*b*-B_{32}/ssDNA at the N/P of 3. Scale bar (A): 100 nm; (B): 500 nm.

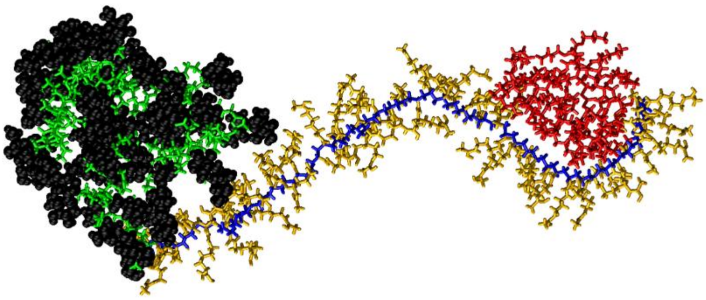

Figure S2. Snapshot from an MD trajectory of M_{62}-*b*-A_{52}-*b*-B_{32} in water (M backbone: green, and mannosyl pendant groups: black; A backbone: blue, and agmatine pendant groups: ochre; B backbone and butyl pendant groups: red).

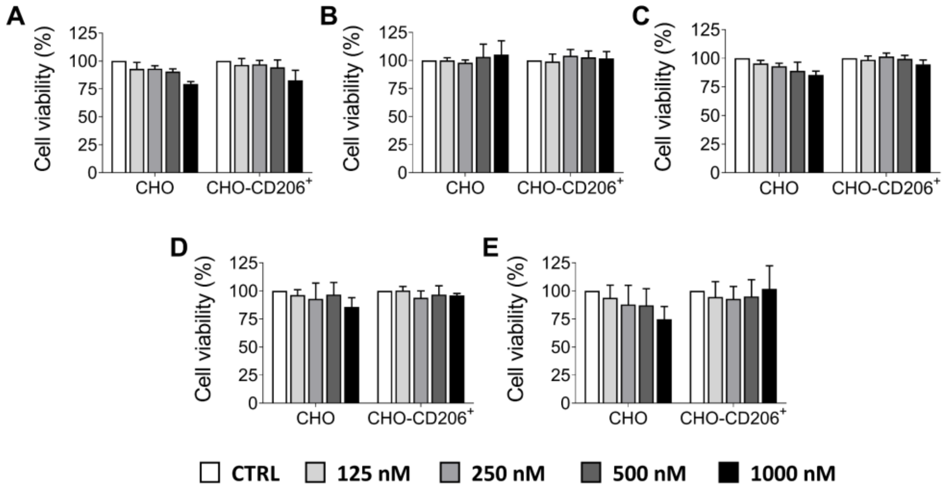

Figure S3. MTT cell viability on CHO and CHO-CD206$^+$ cells. Cells were incubated for 24 h with (A) M_{15}-*b*-A_{12}/ssDNA at the N/P of 20, (B) M_{29}-*b*-A_{25}/ssDNA at the N/P of 10, (C) M_{58}-*b*-A_{45}/ssDNA at the N/P of 5, and (D) M_{29}-*b*-A_{29}-*b*-B_9/ssDNA and (E) M_{62}-*b*-A_{52}-*b*-B_{32}/ssDNA at the N/P of 3 (e) at increasing ssDNA concentrations (0-1000 nM). Three independent experiments were performed in triplicate. Results are reported as mean±s.d (n = 3).

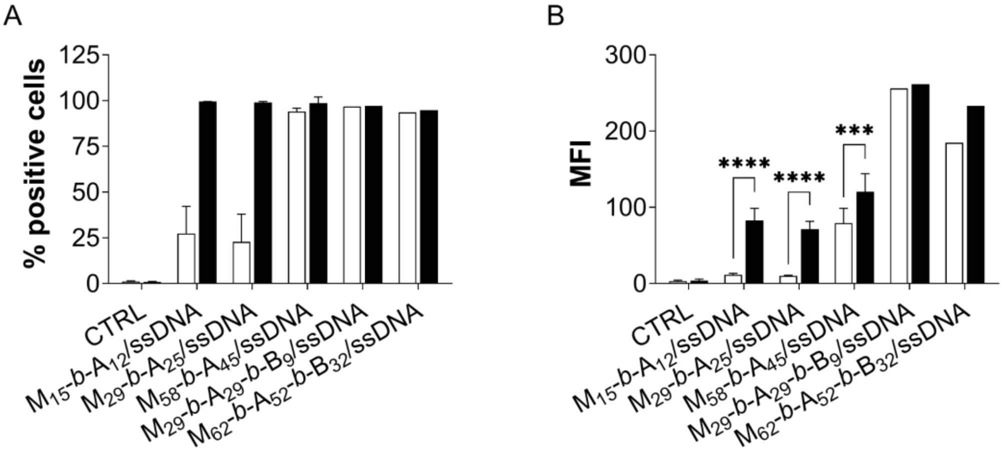

Figure S4. GPPs association profile by flow cytometric (FC) analysis. Percentage of positive cells (A) and the mean fluorescence intensity (MFI) (B) values of CHO (■) and CHO-CD206$^+$ (■) cells at 1 h incubation with 125 nM cy-3 labelled M_{15}-*b*-A_{12}/cy3-ssDNA at the N/P of 20, M_{29}-*b*-A_{25}/cy3-ssDNA at the N/P of 10, M_{58}-*b*-A_{45}/cy3-ssDNA at the N/P of 5, and M_{29}-*b*-A_{29}-*b*-B_9/cy3ssDNA and M_{62}-*b*-A_{52}-*b*-B_{32}/cy3-ssDNA both at the N/P of 3. Data are presented as means ± s.d. (M_x-*b*-A_y/cy3-ssDNA n = 2, ***$p < 0.001$, ****$p < 0.0001$; M_x-*b*-A_y-*b*-B_z/cy3-ssDNA n=1; biological triplicates).

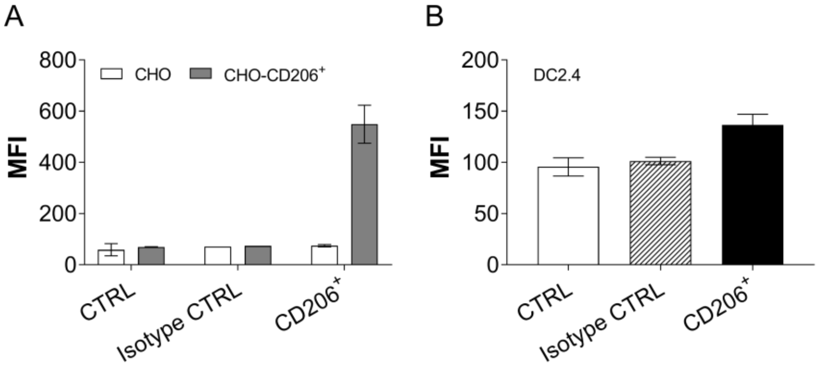

Figure S5. CD206-expression by CHO/CHO-CD206^{+} (A) and DC2.4 (B) cell lines as detected by Flow Cytometry. Unstained cells and cells stained with control isotype Ab were used as controls. MFI: median fluorescence intensity.

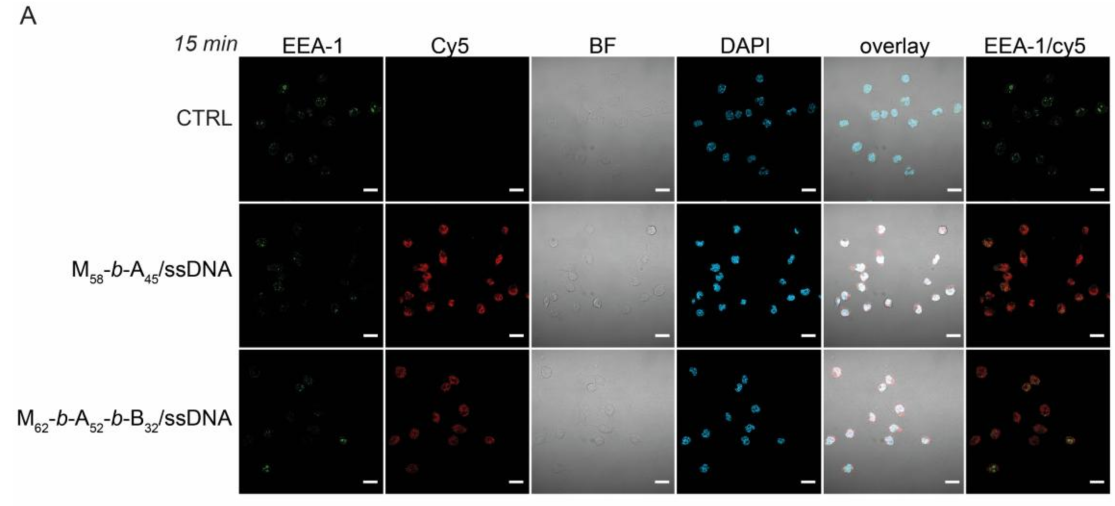
A
15 min
EEA-1
Cy5
BF
DAPI
overlay
EEA-1/cy5
CTRL
M58-b-A45/ssDNA
M62-b-A52-b-B32/ssDNA

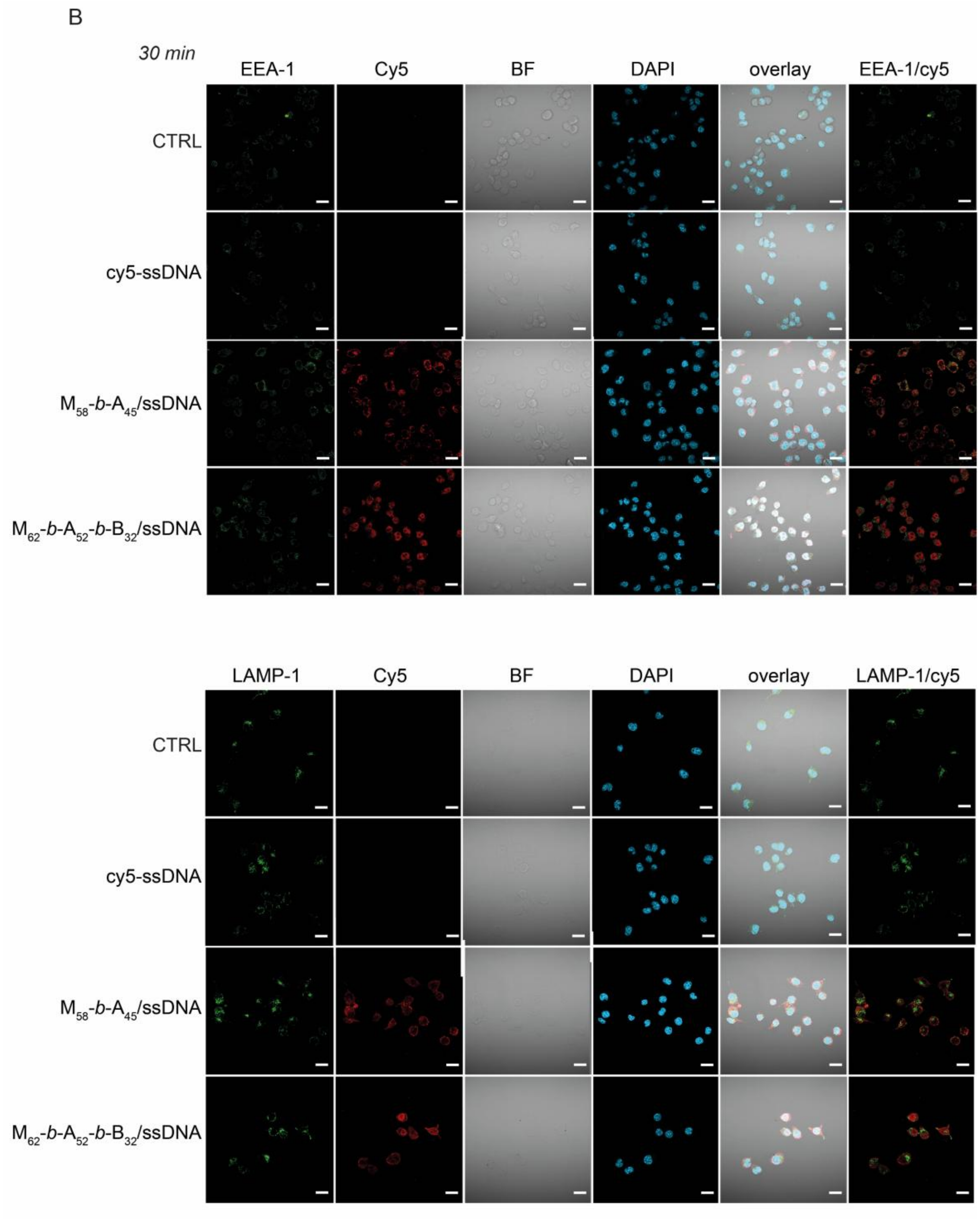

B
30 min
EEA-1
Cy5
BF
DAPI
overlay
EEA-1/cy5
CTRL
cy5-ssDNA
M58-b-A45/ssDNA
M62-b-A52-b-B32/ssDNA
LAMP-1
Cy5
BF
DAPI
overlay
LAMP-1/cy5
CTRL
cy5-ssDNA
M58-b-A45/ssDNA
M62-b-A52-b-B32/ssDNA

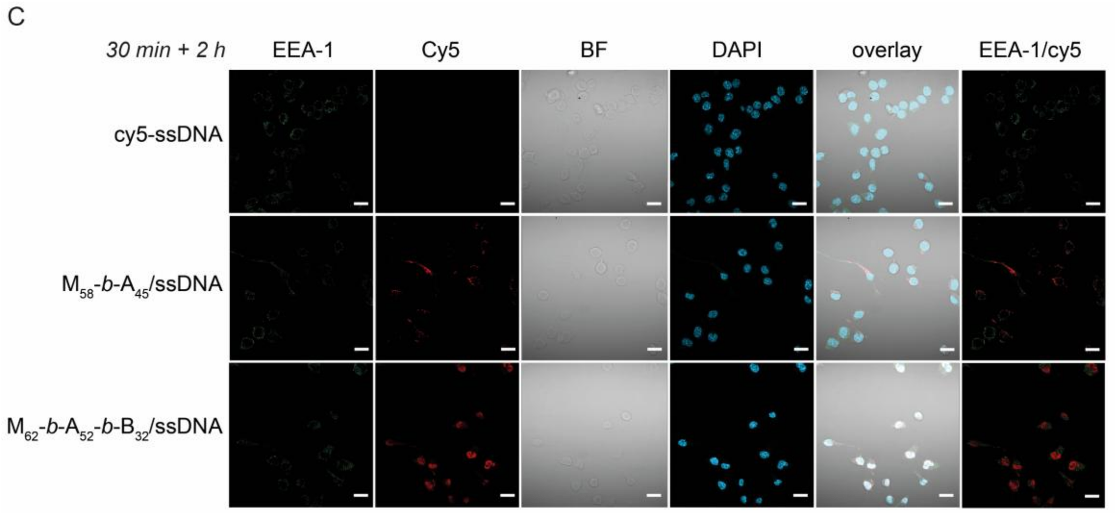

Figure S6. Confocal microscopy images of DC2.4 cell line incubated for (A) 15 or (B) 30 min with M_{58}-*b*-A_{45}/cy5-ssDNA and M_{62}-*b*-A_{52}-*b*-B_{32}/cy5-ssDNA and staining in green for early endosomes (EEA-1) or lysosomes (LAMP-1) (A,B) immediately after incubation or (C) at 2 h post-incubation. Scale bar: 20 µm.

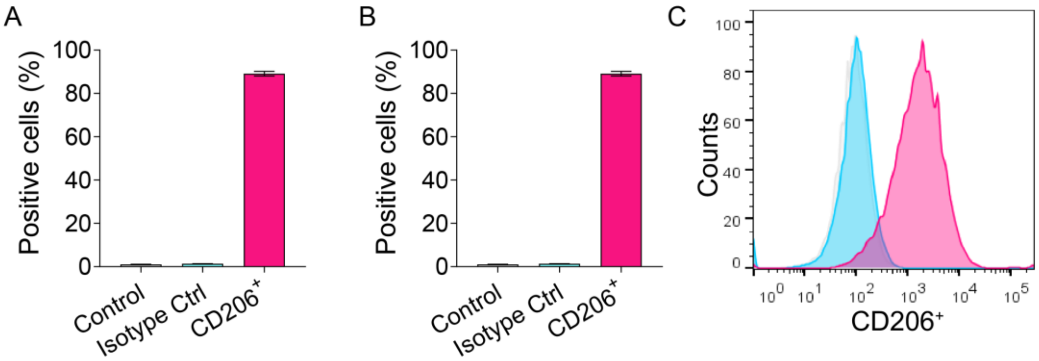

Figure S7. Cell surface CD206 expression by JAWSII cell line. CD206 protein expression on JAWSII was analyzed by flow cytometry with Alexa Fluor® 647 anti-mouse CD206 Antibody. Unstained cells and cells stained with control isotype Ab (Isotype Ctrl) were used as controls. The graphs (from left to right) indicate the percentage of positive cells (A), the median fluorescence intensity (MFI) of the samples (B), and the histogram representation with control cells in light gray, the isotype control-stained cells in blue and the $CD206^{+}$-stained cells in pink (C).

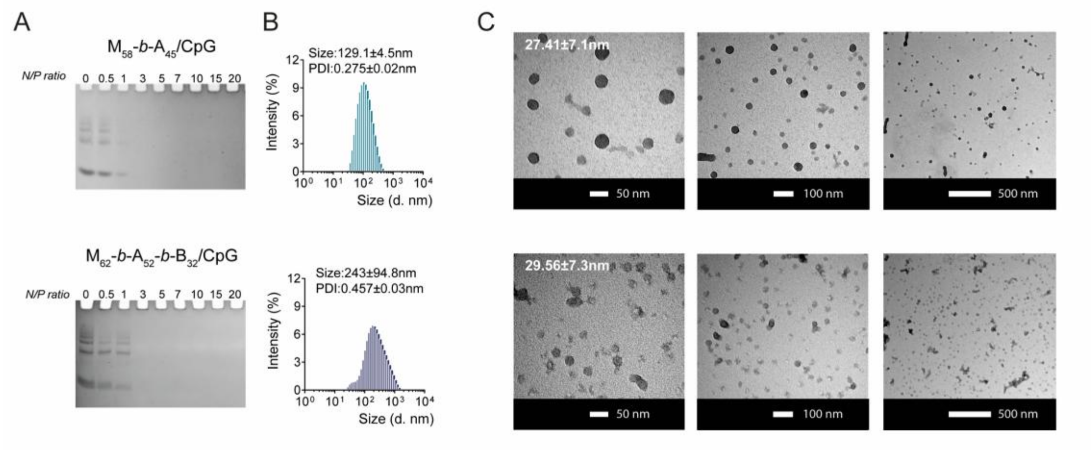

Figure S8. Polymer/CpG characterizations. (A) CpG binding efficiency (complexation) of the polymers in the N/P ratio range of 1-20 was determined by electrophoretic mobility shift assay. (B) DLS profiles and (D) TEM images at different magnifications of M_{58}-*b*-A_{45}/CpG and M_{62}-*b*-A_{52}-*b*-B_{32}/CpG polyplexes.

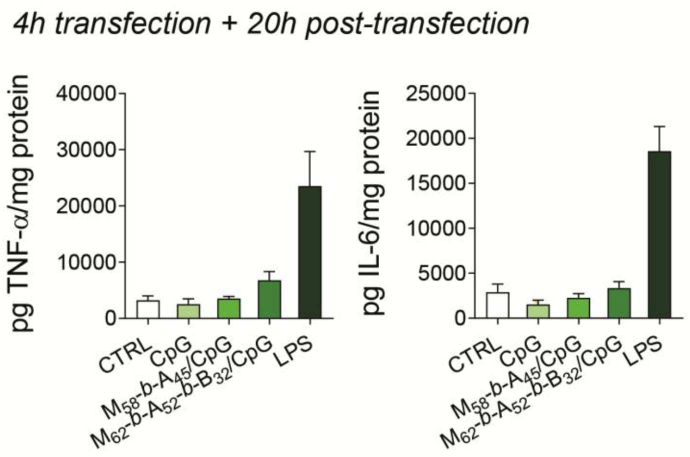

Figure S9. TNF-α and IL-6 cytokines release by JASWII cells induced by 4 h-transfection + 20 h post-transfection with polymers/CpG polyplexes. Untreated cells (CTRL), CpG and LPS alone were used as controls. Data are reported as average ± s.d. of three independent experiments performed in triplicate (*$p < 0.05$, **$p < 0.01$).

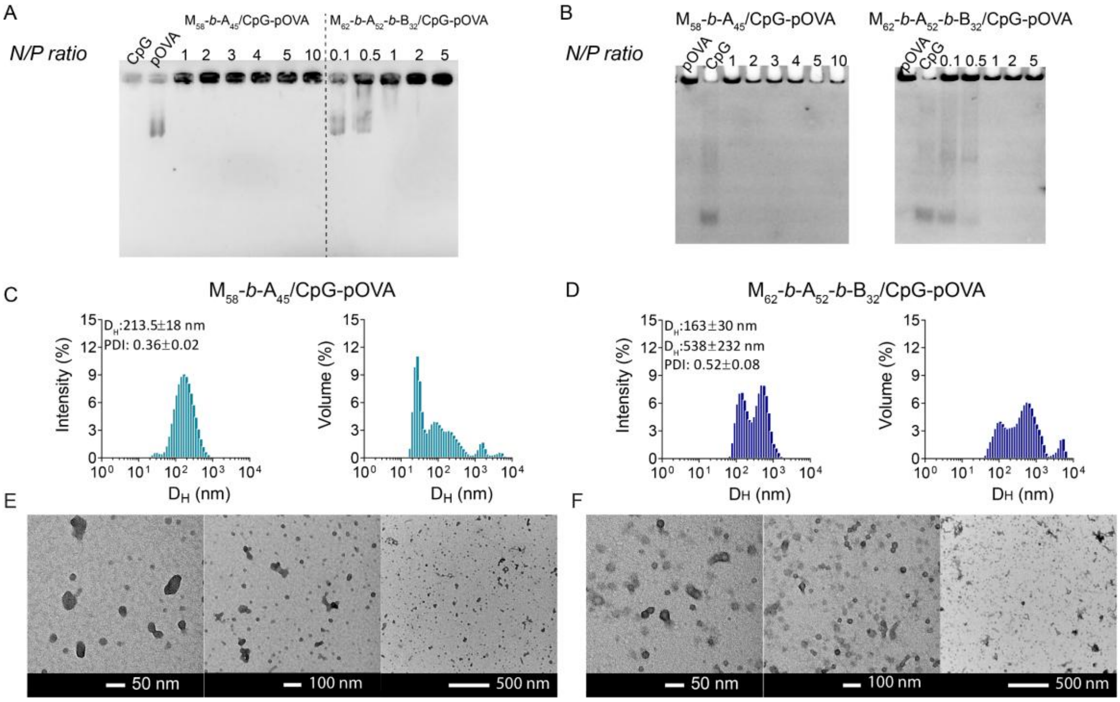

Figure S10. Characterization of M_{58}-*b*-A_{45}/CpG-pOVA and M_{62}-*b*-A_{52}-*b*-B_{32}/CpG-pOVA polyplexes by (A,B) electrophoretic mobility shift assay. (A) 1% agarose gel and (B) 12% acrylamide gel were run before visualizing pOVA and CpG under UV trans-illumination after staining with GelRed. Free pOVA and CpG were used as controls. (C) Dynamic Light Scattering analyses of M_{58}-*b*-A_{45}/CpG-pOVA (C) and M_{62}-*b*-A_{52}-*b*-B_{32}/CpG-pOVA (D) glycoplexes at the N/P ratio of 2. In the case of M_{62}-*b*-A_{52}-*b*-B_{32}/CpG-pOVA a second population was detected by DLS with a size of 538±232 nm. TEM analyses at different magnification of M_{58}-*b*-A_{45}/CpG-pOVA (E) and M_{62}-*b*-A_{52}-*b*-B_{32}/CpG-pOVA (F) glycoplexes.

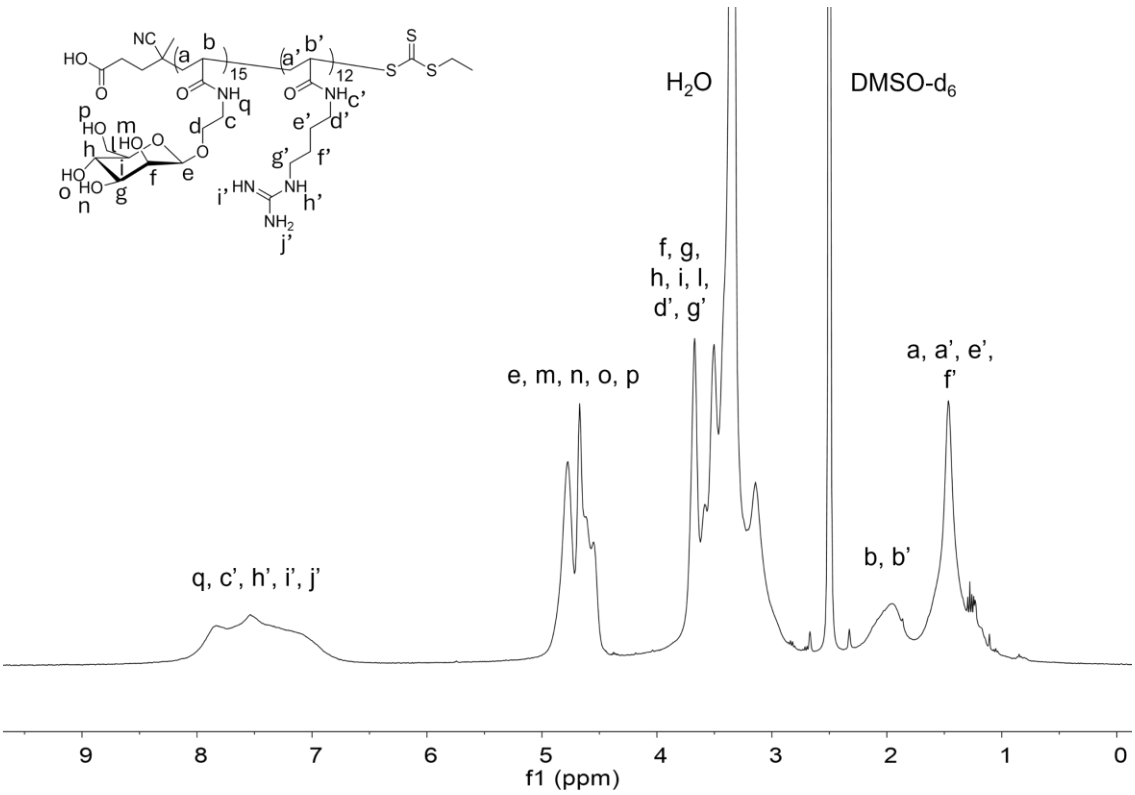

Figure S11. ^{1}H NMR spectrum in DMSO-d_6 of M_{15}-*b*-A_{12} after purification by dialysis and freeze-drying.[1]

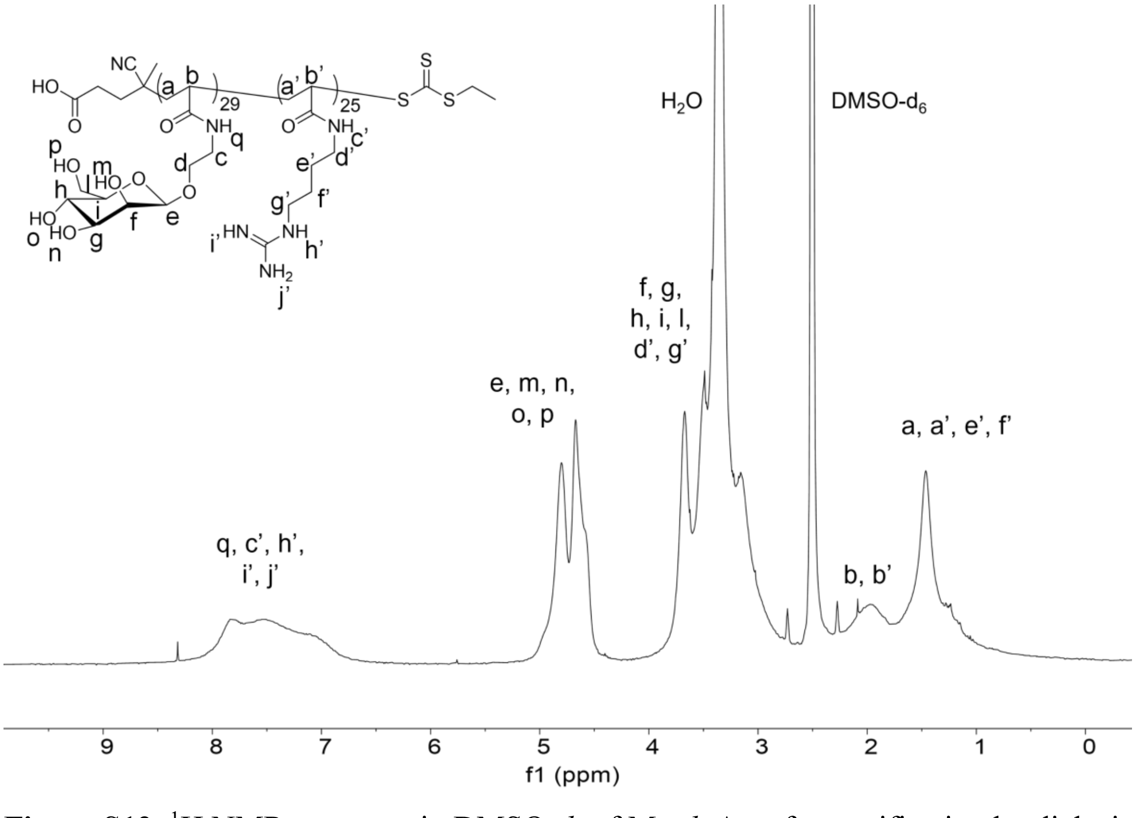

Figure S12. ^{1}H NMR spectrum in DMSO-d_6 of M_{29}-*b*-A_{25} after purification by dialysis and freeze-drying.[1]

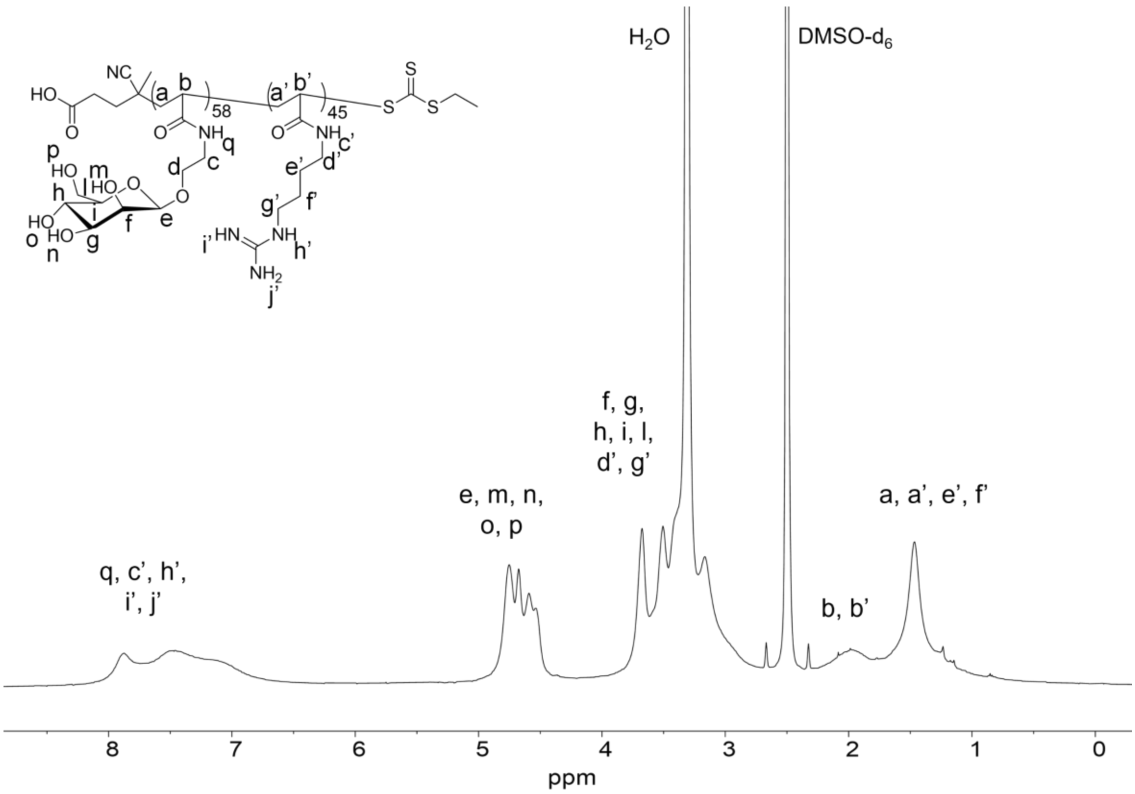

Figure S13. ^{1}H NMR spectrum in DMSO-d_6 of M_{58}-*b*-A_{45} after purification by dialysis and freeze-drying.[1]

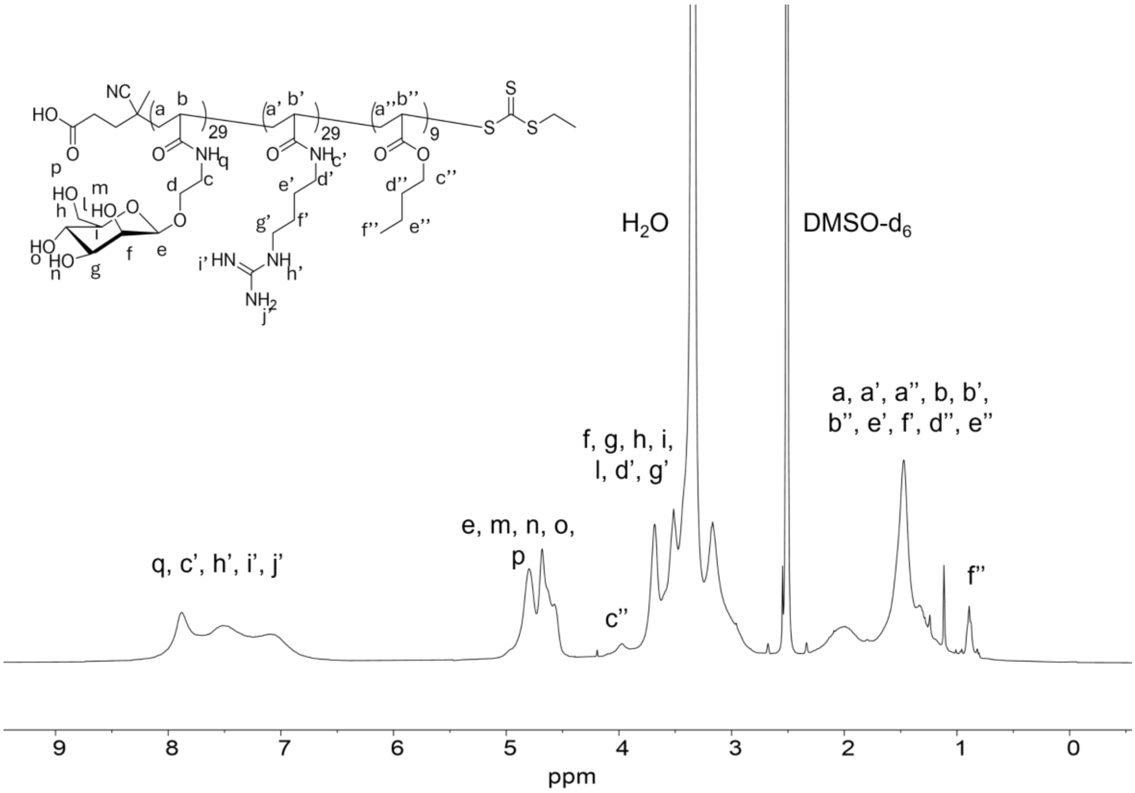

Figure S14. ^{1}H NMR spectrum in DMSO-d_6 of M_{29}-*b*-A_{29}-*b*-B_9 after purification by dialysis and freeze-drying.[1]

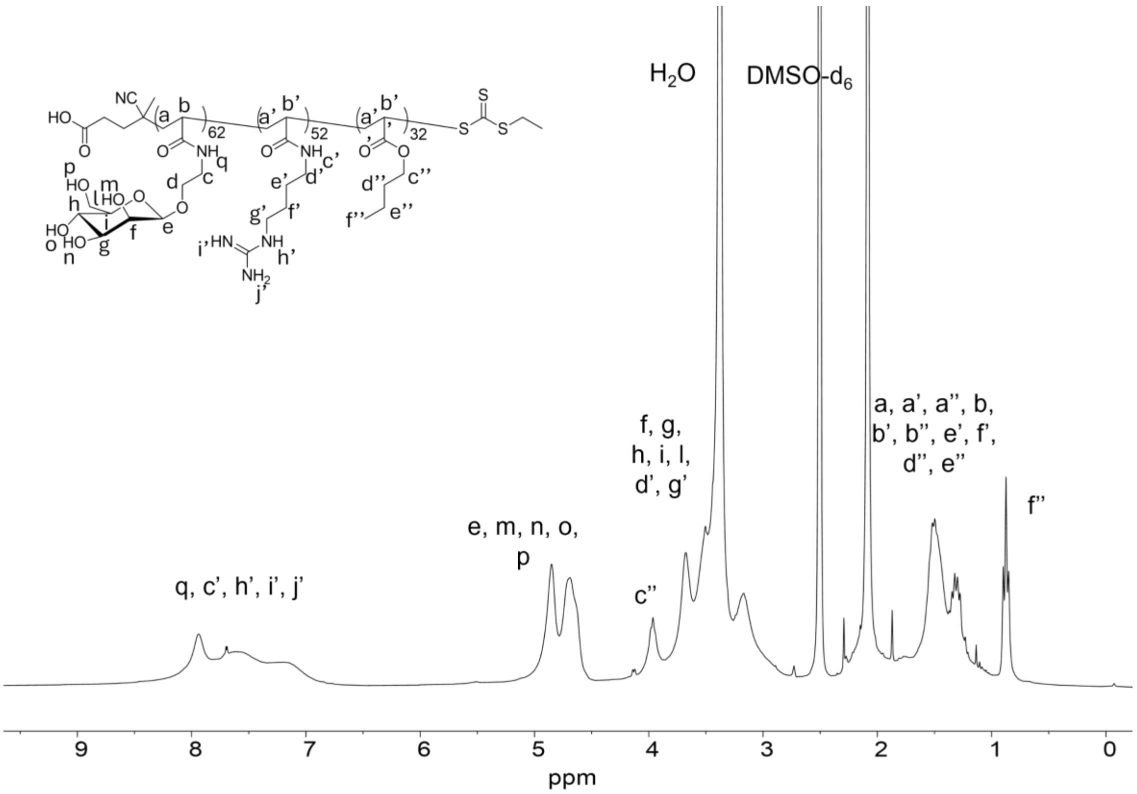

Figure S15. ^{1}H NMR spectrum in DMSO-d_6 of M_{62}-*b*-A_{52}-*b*-B_{32} after purification by dialysis and freeze-drying.[1]

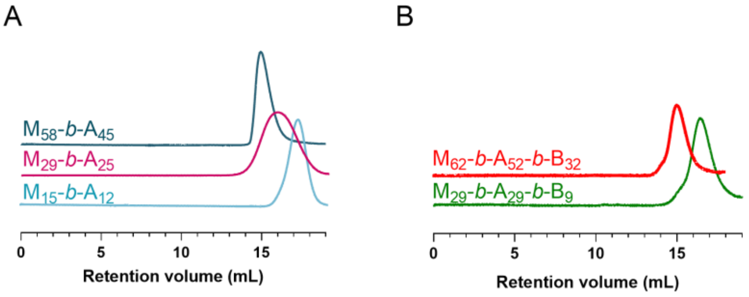

Figure S16. Gel permeation chromatography profiles of (A) M_{15}-*b*-A_{12}, M_{29}-*b*-A_{25}, and M_{58}-*b*-A_{45} di-block copolymers, and (B) M_{29}-*b*-A_{29}-b-B_9, M_{62}-*b*-A_{52}-*b*-B_{32} tri-block copolymers after purification by dialysis and freeze-drying. Samples were analyzed using a Malvern Viscotek TDA302 system (Malvern, UK) equipped with a refractometer (RI), a low-angle light scattering (LALS), a right-angle light scattering (RALS), and a differential viscosimeter (Visc) and thermostated at 40 °C was used. The system was equipped with TOSOH G4000 (10 µm, 7.8 × 300 mm) and G3000 (7 µm, 7.8 × 300 mm)

PWXL columns connected in series and eluted with 0.4 M ammonium acetate buffer, pH 4.5.

References

1. Bellato, F.; Feola, S.; Dalla Verde, G.; Bellio, G.; Pirazzini, M.; Salmaso, S.; Caliceti, P.; Cerullo, V.; Mastrotto, F., Mannosylated Polycations Target CD206+ Antigen-Presenting Cells and Mediate T-Cell-Specific Activation in Cancer Vaccination. *Biomacromolecules* **2022,** *23* (12), 5148-5163.
